# NOGOB receptor deficiency increases cerebrovascular permeability and hemorrhage via impairing histone acetylation–mediated CCM1/2 expression

**DOI:** 10.1172/JCI151382

**Published:** 2022-05-02

**Authors:** Zhi Fang, Xiaoran Sun, Xiang Wang, Ji Ma, Thomas Palaia, Ujala Rana, Benjamin Miao, Louis Ragolia, Wenquan Hu, Qing Robert Miao

**Affiliations:** 1 Department of Foundations of Medicine, New York University Long Island School of Medicine, Mineola, New York, USA.; 2 Department of Surgery and Department of Pathology, Medical College of Wisconsin, Milwaukee, Wisconsin, USA.

**Keywords:** Angiogenesis, Vascular Biology, Endothelial cells, Epigenetics

## Abstract

The loss function of cerebral cavernous malformation (CCM) genes leads to most CCM lesions characterized by enlarged leaking vascular lesions in the brain. Although we previously showed that NOGOB receptor (NGBR) knockout in endothelial cells (ECs) results in cerebrovascular lesions in the mouse embryo, the molecular mechanism by which NGBR regulates CCM1/2 expression has not been elucidated. Here, we show that genetic depletion of *Ngbr* in ECs at both postnatal and adult stages results in CCM1/2 expression deficiency and cerebrovascular lesions such as enlarged vessels, blood-brain-barrier hyperpermeability, and cerebral hemorrhage. To reveal the molecular mechanism, we used RNA-sequencing analysis to examine changes in the transcriptome. Surprisingly, we found that the acetyltransferase HBO1 and histone acetylation were downregulated in *NGBR*-deficient ECs. The mechanistic studies elucidated that NGBR is required for maintaining the expression of CCM1/2 in ECs via HBO1-mediated histone acetylation. ChIP-qPCR data further demonstrated that loss of *NGBR* impairs the binding of HBO1 and acetylated histone H4K5 and H4K12 on the promotor of the *CCM1* and *CCM2* genes. Our findings on epigenetic regulation of CCM1 and CCM2 that is modulated by NGBR and HBO1-mediated histone H4 acetylation provide a perspective on the pathogenesis of sporadic CCMs.

## Graphical abstract

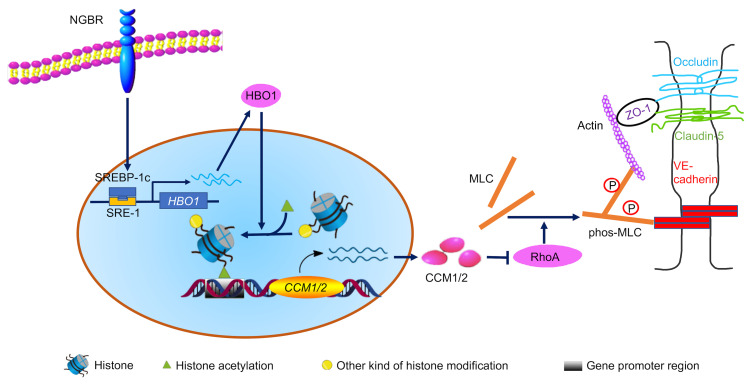

## Introduction

Cerebral cavernous malformations (CCMs) are common vascular malformations in the brain that affect 0.16%–0.9% of the population (1–3). CCMs arise primarily in the endothelium (4–7) and lesions appear in the brain as thin-walled, dilated blood vessels that promote vascular leakage (8–11). As a consequence, CCMs have been implicated as playing a causal role in headaches, seizures, and acute hemorrhagic strokes (12–15). The only treatment currently available for CCMs is neurosurgical lesion resection (16–19). Unfortunately, surgical excision brings a high risk of brain damage, and not all cavernomas can be removed safely (16–18). Loss-of-function mutations of the CCM genes *CCM1*/*KRIT1* (20), *CCM2* (21), and *CCM3*/*PDCD10* (22) at both postnatal and adult stages (4) have been demonstrated in the pathogenesis of CCMs. Based on Knudson’s 2-hit hypothesis in CCM pathogenesis (23–25), the onset of CCMs requires the loss of function/expression of both CCM gene alleles. The first hit would be induced in all cells by a germline mutation that prevents the expression of 1 of the 2 alleles. The second hit would be induced by a somatic mutation or expression loss in the other allele that teams up with the first hit to completely impair CCM gene expression/function (23). Although somatic mutation of CCM genes is also detected in around half of sporadic CCM cases with multiple lesions (26–28), the molecular mechanism responsible for CCM loss in the rest of the sporadic CCM cases has not been elucidated to the best of our knowledge. Our previous study demonstrated that CCM1 protein expression could be decreased under long-term treatment with high glucose, and *Ccm1* heterozygous mice exhibit cerebral hemorrhage under streptozotocin-induced diabetes (29). However, the underlying mechanisms regulating CCM gene expression remain unclear.

HBO1, a member of the MYST lysine acetyltransferase (KAT) family, is also known as histone acetyltransferase 7 (KAT7) and MYST family histone acetyltransferase 2 (MYST2). HBO1 was originally identified as histone acetyltransferase (HAT) binding-origin recognition complex 1 (ORC1) (30–32). Besides its implicated role in DNA replication within ORC1, many pieces of evidence support the idea that HBO1 is involved in transcriptional regulation. HBO1 forms a HAT complex with ING4/5, hEaf6, and JADE1/2/3 or Brpf1/2/3, targeting chromatin by binding lysine residues of histone H3 or H4 (33). The acetyltransferase activity of HBO1 is responsible for the acetylation of histone H4 at lysines 5, 8, and 12 (H4K5, H4K8, and H4K12) (34), and is essential for histone H3 acetylation at lysine 14 (H3K14) (35). These acetylated histone marks at both promoter and intragenic regions mediate cell-restricted gene expression. Altered HBO1 expression has been reported in human abdominal aortic aneurysm (36), a vascular disease closely related to endothelial dysfunction (37). *Hbo1-*knockout mice are embryonic lethal (35). Enlarged blood vessels were noted in the embryonic head, suggesting that HBO1-dependent histone H3K14 acetylation is essential for maintaining normal transcriptional activity during embryonic development (35). HBO1 is engaged in vascular endothelial growth factor receptor 2 (*VEGFR2*) transcription by mediating RNA polymerase II binding and the acetylation of histone H3K14 and histone H4 in the intragenic region of *VEGFR2* (38).

As shown in our previous paper (39), the NOGOB receptor (NGBR) was decreased in the endothelial cells (ECs) of human CCM lesions. Like *Ccm1/2* and *Hbo1* (4, 7, 40), mice with EC knockout of *Ngbr* are embryonic lethal and present enlarged blood vessels in the head region of the embryos. Although we appreciated that NGBR loss results in the downregulation of *CCM1* and *CCM2* expression in ECs, the molecular mechanism by which NGBR regulates the expression of *CCM1* and *CCM2* genes has not been elucidated. Here, we demonstrated the potential link between NGBR loss and CCM pathogenesis by rescuing CCM1/2 expression in mice with inducible EC-specific knockout of *Ngbr* and elucidated the molecular mechanism by which NGBR regulates the transcription of *CCM1/2* genes through HBO1-mediated histone acetylation. We demonstrated that genetic depletion of *Ngbr* in mouse ECs at postnatal and adult stages results in downregulation of CCM1 and CCM2, and consequently blood-brain-barrier (BBB) hyperpermeability and cerebral hemorrhage. Furthermore, these CCM-related lesions can be rescued by adeno-associated virus–mediated (AAV-mediated) gene delivery of either *CCM1/CCM2* or *HBO1*. Mechanistically, NGBR and HBO1 are required to epigenetically regulate the transcription of *CCM1* and *CCM2* genes via HBO1-mediated acetylation of histone H4K5 and H4K12. Our findings provide a perspective on epigenetic regulation of CCM gene transcription in the context of sporadic CCMs. Our data suggest that modulating NGBR- and HBO1-mediated histone H4 acetylation may be an epigenetic regulation related to the pathogenesis of certain types of sporadic CCMs promoted by CCM1/2 deficiency.

## Results

### EC-specific knockout of Ngbr results in hemorrhage and BBB disruption in the brain of postnatal and adult mice.

**Figure 1:**
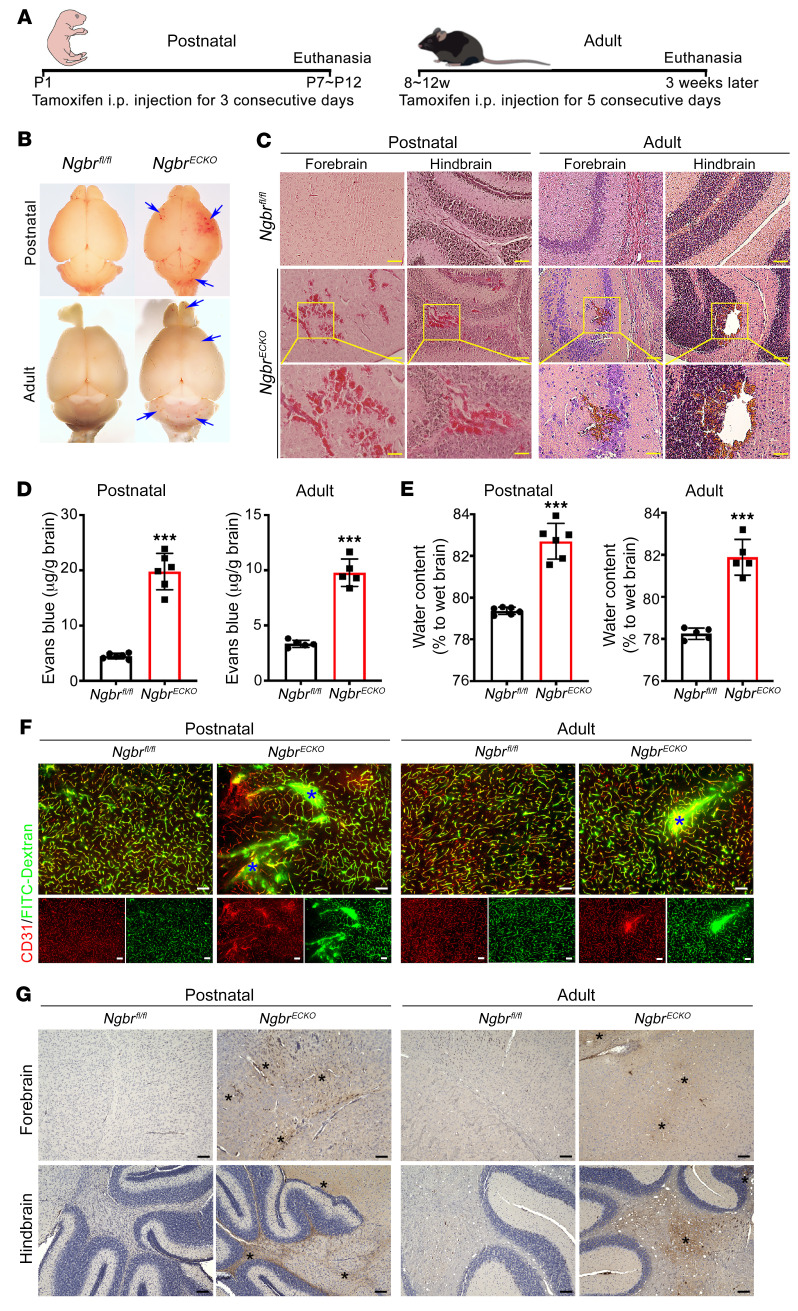
The genetic depletion of *Ngbr* in ECs results in mouse brain hemorrhage and BBB disruption. (**A**) Schedule of intraperitoneal tamoxifen injection in postnatal and adult *Cdh5*-Cre^ERT2^ *Ngbr^fl/fl^* (*Ngbr^ECKO^*) and *Ngbr^fl/fl^* mice at a dose of 75 mg/kg. (**B** and **C**) Representative images of whole-brain fresh tissue from postnatal and adult mice and H&E staining of brain sections. Blue arrows point to bleeding sites (**B**) and bleeding spots observed on H&E images (**C**) in the *Ngbr^ECKO^* group. Scale bars: 100 μm (low-magnification images) and 50 μm (high-magnification images). (**D** and **E**) Evans blue extravasation and brain water content are increased in the brain of postnatal and adult *Ngbr^ECKO^* mice. Data are presented as mean ± SD, *n =* 6 per group for postnatal mice and *n =* 5 per group for adult mice. Significance was tested by 2-tailed, unpaired Student’s *t* test. ***P < 0.001. (**F**) Representative images of immunofluorescent staining on 30-μm FITC-dextran–perfused brain sections showing vessel leakage in the *Ngbr^ECKO^* brain. ECs were labeled by CD31 immunostaining. Blue asterisks indicate the leaking sites. Scale bars: 100 μm. (**G**) Representative IgG staining images show IgG leakage into brain parenchyma in the brain of *Ngbr^ECKO^* mice. Black asterisks indicate leakage sites. Scale bars: 100 μm.

Previously, we and others had shown that EC-specific deletion of *Ngbr* induced embryonic lethality before E12.5 and led to a cerebral vasculogenic defects at the embryonic stage (39, 41). To further address the underlying molecular functions of *Ngbr* in the vasculopathy at the postnatal and adult stage, we conducted EC-specific *Ngbr* knockout in *Cdh5*-Cre^ERT2^ *Ngbr^fl/fl^* mice (hereafter termed *Ngbr^ECKO^*) by intraperitoneal injection of tamoxifen at both the postnatal stage (P1) and the adult stage (8–12 weeks), as illustrated in Figure 1A. *Ngbr^fl/fl^* mice injected with tamoxifen served as littermate controls (hereafter referred to as *Ngbr^fl/fl^*). Consequently, mice at both the postnatal and adult stages developed visible cerebral hemorrhage after the genetic deletion of *Ngbr* in ECs. As shown in Figure 1B, hemorrhage sites were randomly distributed in the brain; however, they were more frequently in the hindbrain. Lesions with red blood cell extravasation were readily detected in hematoxylin and eosin–stained (H&E-stained) sections (Figure 1C). Unlike the postnatal stage, hemorrhage in the brain of adult *Ngbr^ECKO^* mice was not very severe and more like cerebral microbleeds. Hemosiderin staining further indicated stale hemorrhage in adult mice (Supplemental Figure 1A; supplemental material available online with this article; https://doi.org/10.1172/JCI151382DS1).

As the gold-standard confirmatory measurement of BBB disruption, an Evans blue permeability assay showed dramatically increased Evans blue extravasation in the brain of *Ngbr^ECKO^* mice (Figure 1D). Brain edema also was indicated by the significantly increased water content in the brains of *Ngbr^ECKO^* mice compared with *Ngbr^fl/fl^* littermate controls (Figure 1E). FITC-conjugated dextran (FITC-dextran) perfusion showed that FITC-dextran was retained within brain capillary beds of littermate control mice but had infiltrated surrounding tissues around the lesion sites in *Ngbr^ECKO^* mouse brains, as marked with blue asterisks in Figure 1F. When stained with IgG, the *Ngbr^ECKO^* group displayed obvious positive IgG staining around the leaking capillaries, as marked with black asterisks in Figure 1G. These defects were observed in the brain of both postnatal and adult *Ngbr^ECKO^* mice.

### Ngbr^ECKO^ mouse brains exhibit enlarged microvessels, increased phosphorylation of MLC, and disrupted adherens junctions and tight junctions.

**Figure 2:**
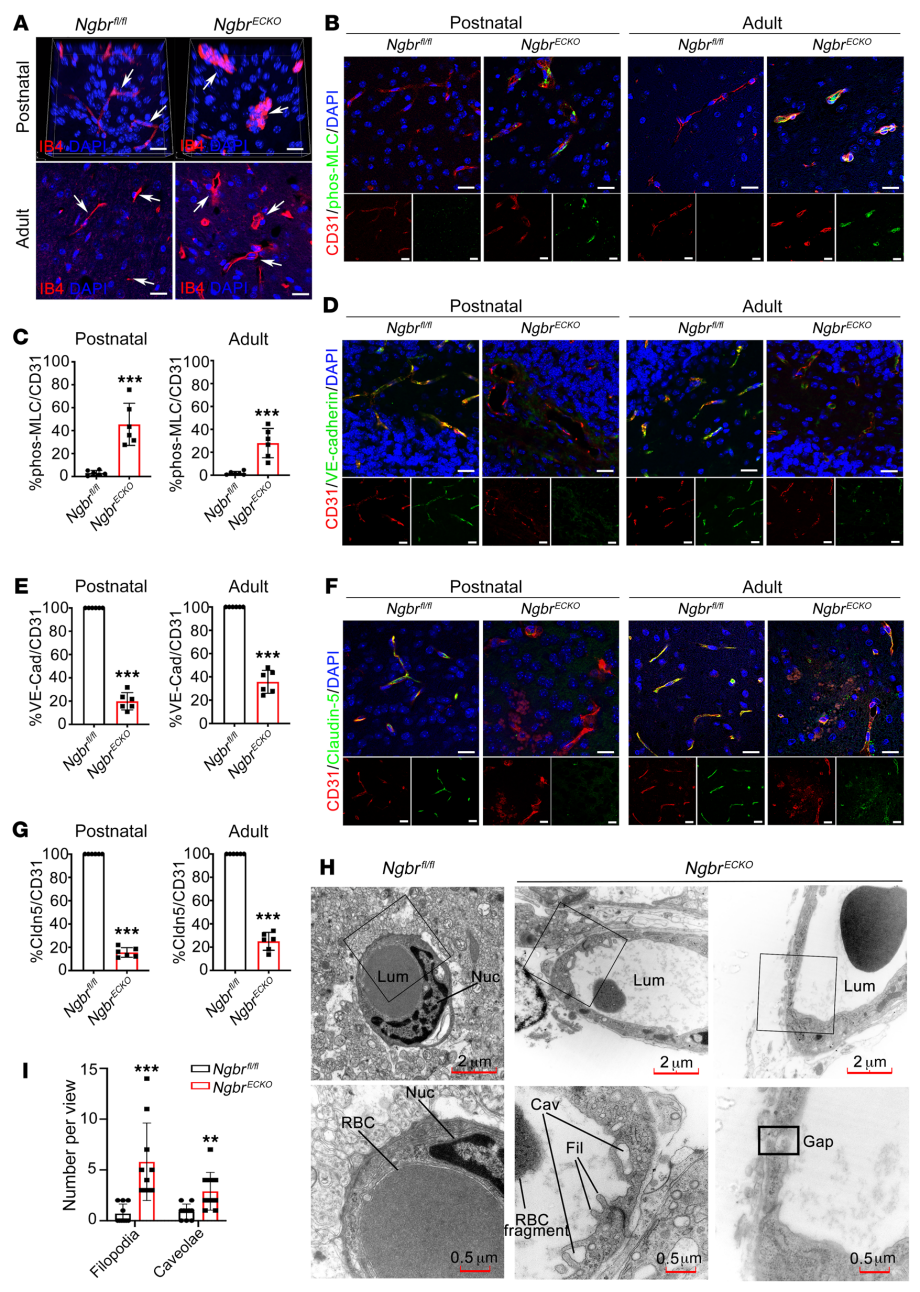
EC-specific *Ngbr*-knockout mice exhibit enlarged microvessels, increased phosphorylation of MLC, and disrupted AJs and TJs in the brain. (**A**) Immunofluorescent staining showing enlargement of microvessels in the brain of *Ngbr^ECKO^* mice. IB4 (red) was used to label ECs and DAPI (blue) was used to label nuclei. Scale bars: 20 μm. (**B**–**G**) Immunofluorescent staining and quantification results showing increased phos-MLC and impaired AJs (VE-cadherin, VE-cad) and TJs (claudin-5, Cldn5) coverage of ECs in the brain lesions of *Ngbr^ECKO^* mice. Tissue sections were stained for phos-MLC (green)/CD31 (red)/DAPI (blue), claudin-5 (green)/CD31 (red)/DAPI (blue), or VE-cadherin (green)/CD31 (red)/DAPI (blue). Scale bars: 20 μm (**B**, **D**, and **F**). Results were quantified using ImageJ software. Data are presented as mean ± SD, *n =* 6 per group . Significance was tested by 2-tailed, unpaired Student’s *t* test. ***P < 0.001 (**C**, **E**, and **G**). (**H** and **I**) Electron microscopy images of microvessels in the brain and quantification of filopodia (Fil) and caveolae (Cav) in both *Ngbr^fl/fl^* and *Ngbr^ECKO^* groups. Electron microscopy images show enlarged vessel lumen (Lum) and irregular endothelial shape with a rough luminal surface in the brain of *Ngbr^ECKO^* mice compared with that of *Ngbr^fl/fl^* mice. Caveolae, filopodia, and junction gaps (Gap) between ECs were observed in the *Ngbr^ECKO^* group but not in the littermate control group (*Ngbr^fl/fl^*). RBC, red blood cells; Nuc, nucleus. Scale bars: 2 μm (low-magnification images and 0.5 μm (high-magnification images). *n =* 10 views randomly selected from 3 mice per group. Significance was tested by 2-tailed, unpaired Student’s *t* test. **P < 0.01, ***P < 0.001.

To determine vascular morphology changes in the *Ngbr^ECKO^* mouse brain, mouse brain and retina sections were stained with the EC marker isolectin B4 (IB4). As shown in Figure 2A, Supplemental Figure 1, B and C, and Supplemental Videos 1 and 2, brain and retina microvessels were markedly enlarged in the lesion sites of *Ngbr^ECKO^* mice compared with *Ngbr^fl/fl^* mice. In *Ngbr^ECKO^* mouse brains, phosphorylation of MLC (phos-MLC) was induced in the ECs of lesion sites (Figure 2, B and C), suggesting a change in EC contractility. Moreover, immunofluorescent staining results showed a significant decrease in adherens junctions (AJs, represented by green VE-cadherin staining) and tight junctions (TJs, represented by green claudin-5 staining) between ECs in the lesions of *Ngbr^ECKO^* mouse brains (Figure 2, D–G). These junctional defects contributed to disrupted vessel integrity and increased BBB permeability observed in the brain of *Ngbr^ECKO^* mice, as shown in Figure 1. Electron microscopy images and quantification further revealed changes in the ECs in *Ngbr^ECKO^* mouse brains (Figure 2, H and I). An elongated shape and giant nuclei of ECs and a rough luminal surface of capillaries were noted in the *Ngbr^ECKO^* group compared with that of *Ngbr^fl/fl^* controls. Intracytoplasmic canaliculi (caveolae), protoplasmic protrusions (filopodia), and gaps between ECs were observed in the *Ngbr^ECKO^* group but not in the *Ngbr^fl/fl^* control group. These findings indicate that *Ngbr* deficiency promotes the activation of ECs.

### Ngbr deficiency diminishes the expression of CCM1 and CCM2 in brain ECs.

The enlarged microvessels and cerebral hemorrhage phenotype in the brain of *Ngbr^ECKO^* mice were similar to vascular defects presented in the brain of EC-specific *Ccm1-* or *Ccm2*-knockout mice (4, 7, 40). Our previous work showed that NGBR was decreased in human CCM lesions, and *Ccm1/2* was suppressed in *Ngbr*-deficient yolk sac (39). The pathological changes of brain ECs in *Ngbr^ECKO^* mice are identical to that of ECs from human CCM lesions (9–11), such as increased RhoA/phos-MLC signaling and junctional disruption (42–44).

**Figure 3:**
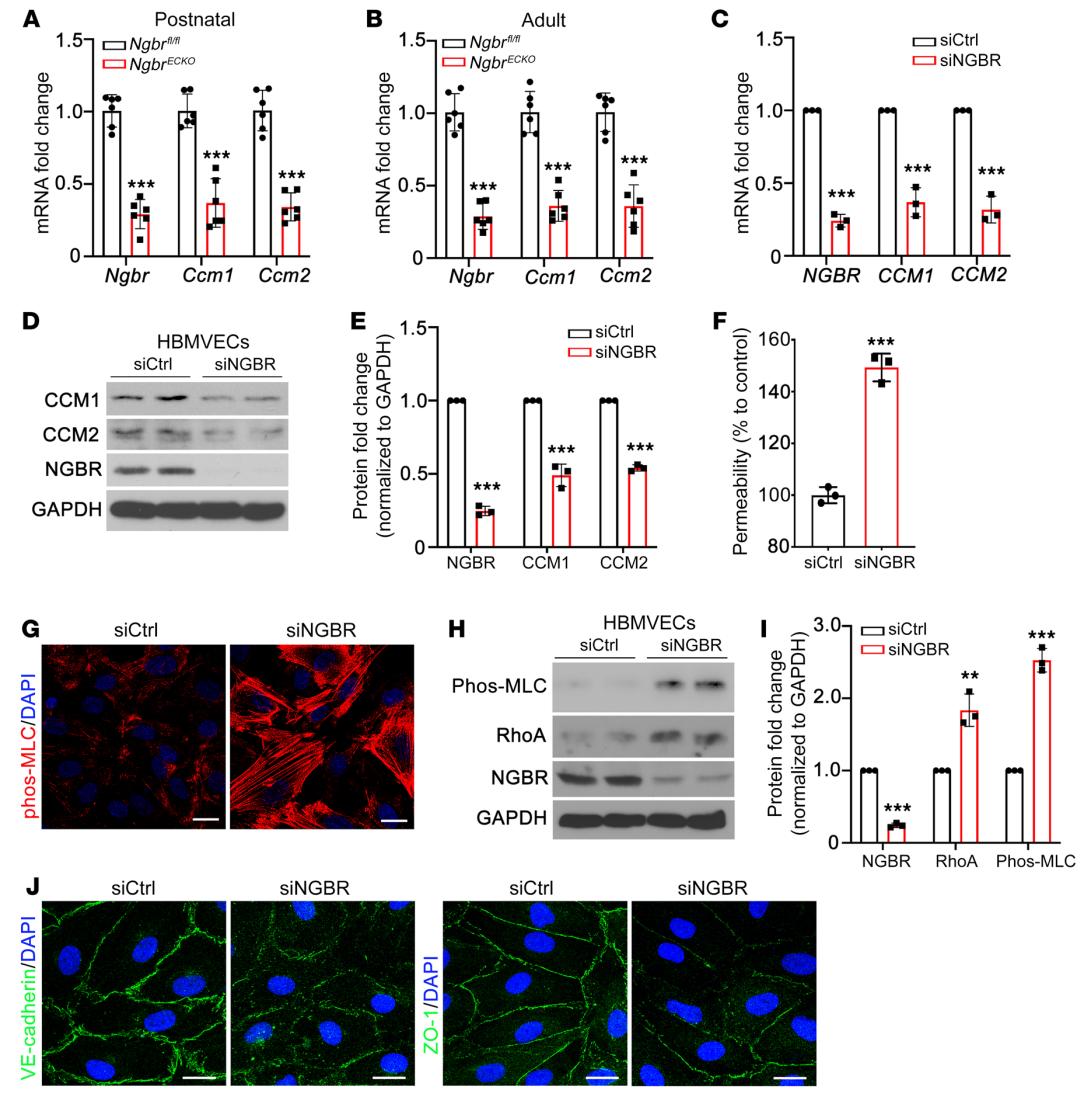
*NGBR* deficiency decreases CCM1/2 expression, increases endothelial permeability, and promotes RhoA/phos-MLC signaling. (**A**–**C**) *CCM1* and *CCM2* mRNA levels decreased in both MBMVECs and HBMVECs due to *NGBR* deficiency. (**A** and **B**) ECs extracted from postnatal and adult-stage mouse brains (MBMVECs) after tamoxifen injection. (**C**) *NGBR* in HBMVECs was knocked down with siRNA. mRNA levels were determined by RT-qPCR. siCtrl, control siRNA–treated group; siNGBR, *NGBR* siRNA–treated group. (**D** and **E**) Western blot and quantification results showing that CCM1 and CCM2 protein levels decrease in *NGBR*-deficient HBMVECs. (**F**) *NGBR* deficiency results in an increase in endothelial permeability as determined by EC-monolayer permeability assay. (**G**) Immunofluorescent staining showing increased phos-MLC in *NGBR*-deficient HBMVECs. Scale bars: 10 μm. (**H** and **I**) Western blot and quantification results showing RhoA and phos-MLC increases in *NGBR*-knockdown HBMVECs. (**J**) Immunofluorescent staining showing the impaired AJs (VE-cadherin) and TJs (ZO-1) in *NGBR*-deficient HBMVECs. Scale bars: 10 μm. Data are presented as mean ± SD, *n =* 6 mice per group (**A** and **B**) and *n =* 3 samples per group (**C**, **E**, **F**, and **I**). **P < 0.01, ***P < 0.001. Significance was tested by 2-tailed, unpaired Student’s *t* test (**A**–**C**, **E**, **F**, and **I**).

To test the hypothesis that NGBR is required for regulating the expression of CCM genes in ECs, we extracted mouse brain microvascular ECs (MBMVECs) from both postnatal and adult *Ngbr^ECKO^* mice as well as littermate control mice. As compared with *Ngbr^fl/fl^* MBMVECs, the expression of *Ccm1* and *Ccm2* but not *Ccm3* was markedly decreased in *Ngbr*-deficient MBMVECs isolated from the brain of either postnatal or adult *Ngbr^ECKO^* mice (Figure 3, A and B, and Supplemental Figure 2A). In human brain microvascular ECs (HBMVECs) in vitro, *NGBR* was knocked down with *NGBR* siRNA, the efficacy of which has been optimized and validated in our previous publications (45). The mRNA and protein levels of CCM1 and CCM2, but not CCM3, were also significantly decreased in *NGBR*-deficient HBMVECs (Figure 3, C–E, and Supplemental Figure 2, B and C).

Consistent with the in vivo study, *NGBR* knockdown in HBMVECs significantly increased the monolayer permeability (Figure 3F), increased RhoA/phos-MLC signaling (Figure 3, G–I), and impaired the formation of AJs (represented by VE-cadherin staining) and TJs (represented by ZO-1 staining) (Figure 3J). Like *NGBR* deficiency, knockdown of either *CCM1* or *CCM2* in HBMVECs resulted in increased permeability (Supplemental Figure 3A). As shown in Supplemental Figure 3B, immunostaining signals of phos-MLC were increased in *CCM1*- and *CCM2*-knockdown HBMVECs. Correspondingly, knockdown of either *CCM1* or *CCM2* increased the RhoA protein levels in HBMVECs (Supplemental Figure 3, C and D). However, *CCM1* or *CCM2* knockdown did not affect NGBR expression in HBMVECs (Supplemental Figure 3, C and D), suggesting that NGBR is an upstream regulator of CCM1 and CCM2.

### Overexpression of CCM1 and CCM2 ameliorates Ngbr deficiency–promoted vascular defects in vitro and in vivo.

**Figure 4:**
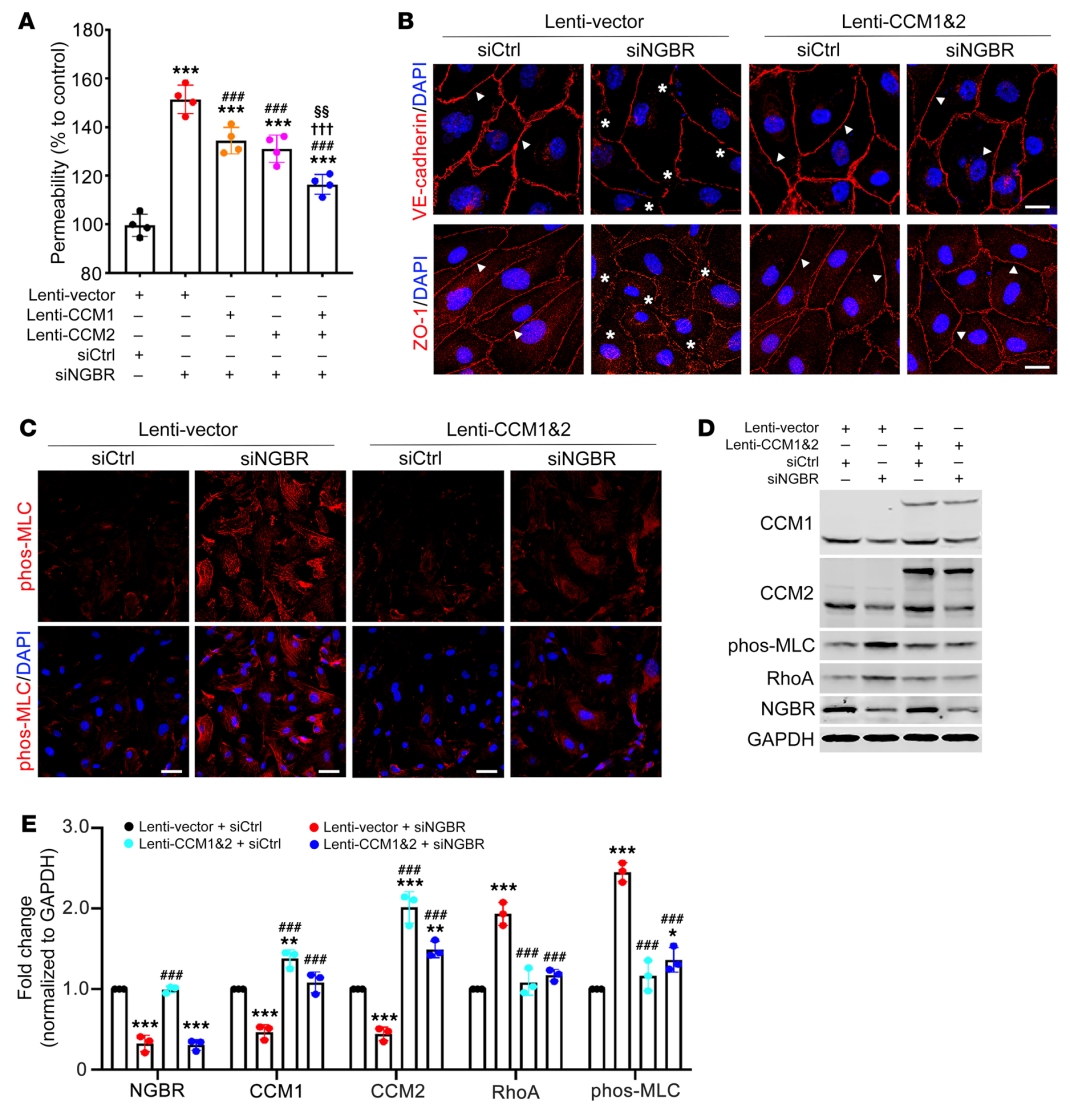
*CCM1/2* overexpression ameliorated *NGBR* deficiency–induced endothelial dysfunction in vitro. (**A**) The overexpression of *CCM1* and *CCM2* genes using lentiviruses ameliorated *NGBR* deficiency–promoted HBMVEC hyperpermeability, which was determined by EC-monolayer permeability assay. Statistical significance was determined by 1-way ANOVA and Dunnett’s post hoc test. Data are presented as mean ± SD. ***P < 0.001 versus siCtrl- and lentivirus vector control–treated (lenti-vector) group; ^###^*P* < 0.001 versus siNGBR- and lenti-vector–treated group; ^†††^*P* < 0.001 versus siNGBR- and CCM1 lentivirus–treated (lenti-CCM1) group; ^§§^*P* < 0.01 versus siNGBR- and CCM2 lentivirus–treated (lenti-CCM2) group. *n =* 4 samples per group. (**B**) Immunofluorescent staining showing that overexpression of *CCM1* and *CCM2* synergistically improved *NGBR* deficiency–induced AJ (VE-cadherin) and TJ (ZO-1) disruption. Scale bars: 10 μm. (**C**) Overexpression of *CCM1* and *CCM2* genes resulted in a synergistic decrease in phos-MLC immunofluorescent staining in *NGBR*-deficient HBMVECs. Scale bars: 20 μm. (**D** and **E**) Western blot and quantification results showing that overexpression of *CCM1* and *CCM2* synergistically diminished the induction of RhoA and phos-MLC in *NGBR*-deficient HBMVECs. Data are presented as mean ± SD, *n =* 3 samples per group. Statistical significance was determined by 1-way ANOVA with Dunnett’s post hoc test. *P < 0.05, **P < 0.01, ***P < 0.001 versus control siRNA–and lenti-vector–treated HBMVECs; ^###^*P* < 0.001 versus *NGBR* siRNA– and lenti-vector–treated HBMVECs.

To further confirm the contributions of CCM1 and CCM2 downregulation to the pathogenesis of *NGBR*-deficient ECs, we generated lentiviruses harboring either the *CCM1* or *CCM2* transgene and confirmed the overexpression of CCM1 and CCM2 proteins in HBMVECs in vitro. As shown in Supplemental Figure 4, A and B, the efficiency of lentivirus-mediated overexpression of CCM1 and CCM2 was sufficient. The overexpression of *CCM1* and *CCM2* did not affect NGBR expression in HBMVECs. As shown in Figure 4A, overexpression of either *CCM1* or *CCM2* reduced the hyperpermeability of *NGBR*-deficient HBMVECs and showed synergistic effects in reducing the hyperpermeability to a greater extent when overexpressing both *CCM1* and *CCM2*. Therefore, we used *CCM1* and *CCM2* overexpression for the following rescue experiments. As a result, overexpression of *CCM1* and *CCM2* eliminated the defects in AJs (VE-cadherin) and TJs (ZO-1) (Figure 4B) and diminished the induction of phos-MLC in *NGBR*-deficient HBMVECs (Figure 4C). The quantitative changes in RhoA and phos-MLC were further determined by Western blotting analysis (Figure 4, D and E). These results show that *CCM1* and *CCM2* overexpression suppresses the induction of RhoA and phos-MLC in *NGBR*-deficient HBMVECs.

**Figure 5:**
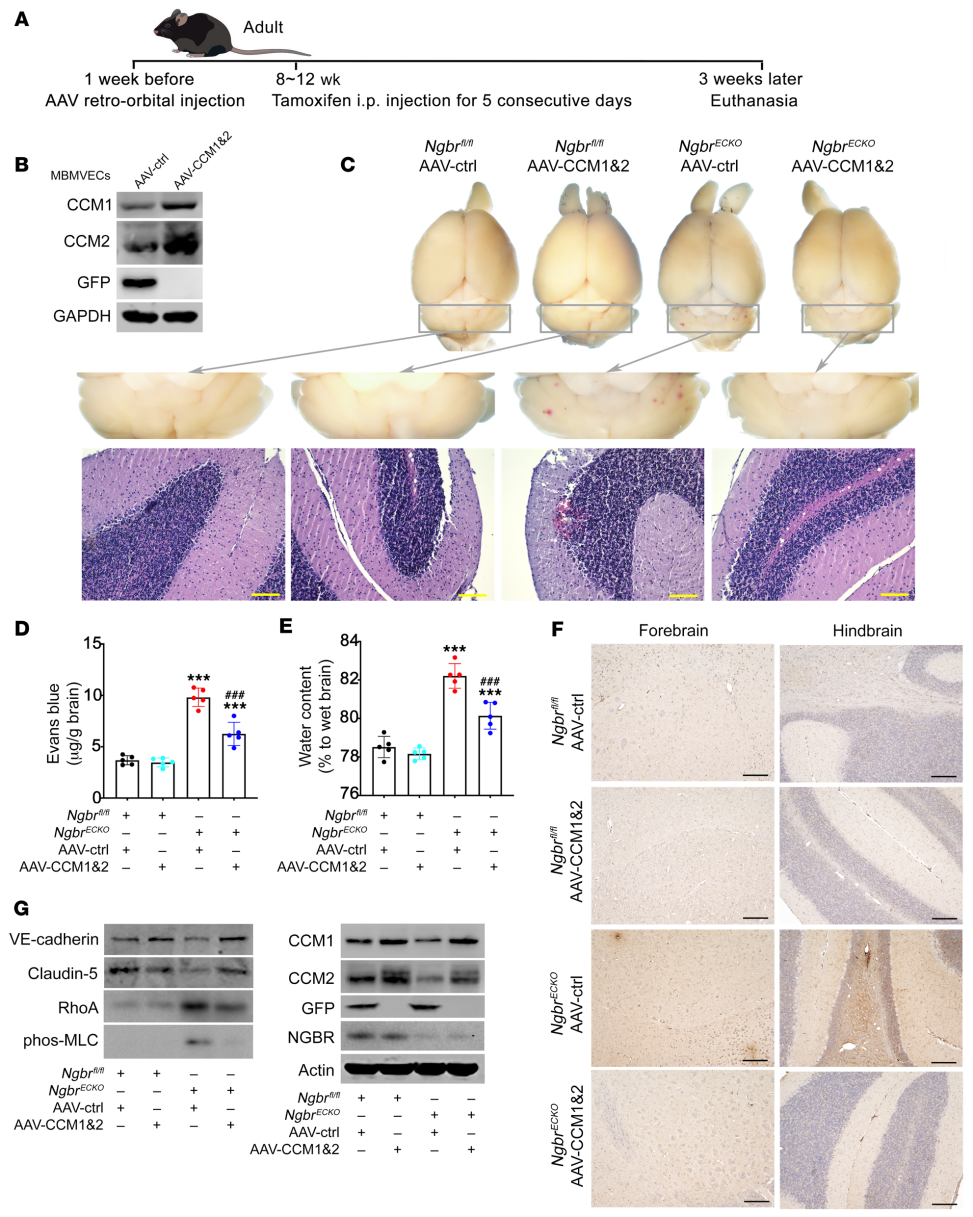
The overexpression of CCM1 and CCM2 in brain ECs diminishes *Ngbr* deficiency–promoted microvessel leakage and hemorrhage in vivo. CCM1 and CCM2 overexpression in brain ECs was achieved by AAV-CCM1 and AAV-CCM2 administration. AAV-BR1-GFP was used as control (AAV-ctrl). (**A**) Tamoxifen was injected 1 week after AAV injection, and mice were euthanized 3 weeks after tamoxifen injection as shown in the diagram protocol. (**B**) The efficacy of AAV-BR1–mediated overexpression of CCM1 and CCM2 was determined by Western blotting of the lysates of MBMVECs extracted from the brain of mice injected with AAV-CCM1 and -2 or AAV-ctrl. The results demonstrated sufficient overexpression of CCM1 and CCM2 in MBMVECs in vivo. (**C**) Representative images of hemorrhage in fresh brain tissues and H&E staining. Hemorrhage sites were observed in the brain of AAV-ctrl–injected *Ngbr^ECKO^* mice. In contrast, scarce hemorrhage sites were observed in the brain of the AAV-CCM1/2–injected *Ngbr^ECKO^* group. Scale bars: 100 μm. (**D** and **E**) CCM1 and CCM2 overexpression significantly diminished the *Ngbr* deficiency–promoted hyperpermeability, determined by calculation of Evans blue extravasation and brain water content. Data are presented as mean ± SD, *n =* 5 mice per group. Significance was tested by 1-way ANOVA with Dunnett’s post hoc test. ***P < 0.001 versus *Ngbr^fl/fl^* mice treated with AAV-ctrl; ^###^*P* < 0.001 versus *Ngbr^ECKO^* mice treated with AAV-ctrl. (**F**) IgG staining showing increased IgG-positive staining in AAV-ctrl–injected *Ngbr^ECKO^* mice compared with AAV-ctrl–injected *Ngbr^fl/fl^* mice, while significantly decreased IgG-positive staining was observed in AAV-CCM1/2–injected *Ngbr^ECKO^* mice. Scale bars: 200 μm. (**G**) Western blotting was used to determine the protein levels in MBMVECs extracted from 5 mice in each group. Results showed that overexpression of *CCM1* and *CCM2* genes mitigated the hyperactivation of RhoA/phos-MLC signaling and the impairment of AJs (VE-cadherin) and TJs (claudin-5) in MBMVECs of *Ngbr^ECKO^* mice.

The rescue effects of *CCM1* and *CCM2* overexpression were further confirmed in vivo. As reported by Korbelin et al. and Dogbevia et al., AAV serotype BR1 (AAV-BR1) specifically targets brain ECs and has been used for brain EC–specific gene overexpression (46, 47). We generated AAV-BR1 harboring either *CCM1* (AAV-BR1-CCM1, AAV-CCM1) or *CCM2* (AAV-BR1-CCM2, AAV-CCM2) genes. AAV-BR1 expressing *GFP* (AAV-BR1-GFP) was used as a control (AAV-ctrl). As shown in Supplemental Figure 5, the efficiency and tissue specificity of AAV-BR1-GFP expression were determined by examining GFP expression in various tissues. At 2 weeks after tail vein injection of AAV-BR1-GFP, positive GFP staining was exclusively present in brain ECs but not in any ECs in the heart, lung, liver, and kidney. A schematic of the protocol for animal treatment is shown in Figure 5A. For in vivo rescue experiments, 1 week after AAV-BR1 injection, tamoxifen administration was used to induce genetic deletion of *Ngbr* in ECs. The overexpression of *CCM1* and *CCM2* was detected in MBMVECs extracted from the brains of mice injected with both AAV-BR1-CCM1 and AAV-BR1-CCM2 compared with AAV-ctrl–injected mice (Figure 5B). As shown in Figure 5C, *CCM1* and *CCM2* overexpression reduced the hemorrhagic lesions in the brain of *Ngbr^ECKO^* mice, as visualized by whole-mount brain images and H&E staining. As shown in Figure 5, D and E, *CCM1* and *CCM2* overexpression in the brain ECs of *Ngbr^ECKO^* mice remarkably attenuated the BBB leakage and brain edema, as determined by Evans blue extravasation assay and water content measurement, respectively. The reduced IgG staining further confirmed the effects of *CCM1* and *CCM2* overexpression on rescuing the BBB integrity of *Ngbr^ECKO^* mice (Figure 5F). Western blotting results and immunofluorescence staining showed the restoration of AJs (VE-cadherin) and TJs (claudin-5) protein levels, as well as a reduction in phos-MLC in mouse brain ECs (Supplemental Figure 6, A–C) and MBMVECs extracted from the brain of *Ngbr^ECKO^* mice overexpressing *CCM1* and *CCM2* (Figure 5G). These results indicated that downregulation of CCM1 and CCM2 can be attributed to the *Ngbr* deficiency–promoted BBB disruption and cerebral hemorrhage.

### HBO1-mediated histone acetylation is essential for NGBR deficiency–promoted transcriptional downregulation of CCM1 and CCM2 in HBMVECs.

Since NGBR is the receptor for soluble NOGOB (sNOGOB), we detected NOGOB expression in *Ngbr^ECKO^* MBMVECs and plasma concentration of sNOGOB in *Ngbr^ECKO^* mice. The results showed that EC *Ngbr* knockout neither influences NOGOB expression in MBMVECs nor the serum concentration of sNOGOB (Supplemental Figure 7, A and B). Consistently, *NGBR* knockdown did not affect the NOGOB expression in HBMVECs (Supplemental Figure 7C). These results indicate that NGBR-mediated CCM1/2 expression regulation is independent of the alteration of NOGOB.

**Figure 6:**
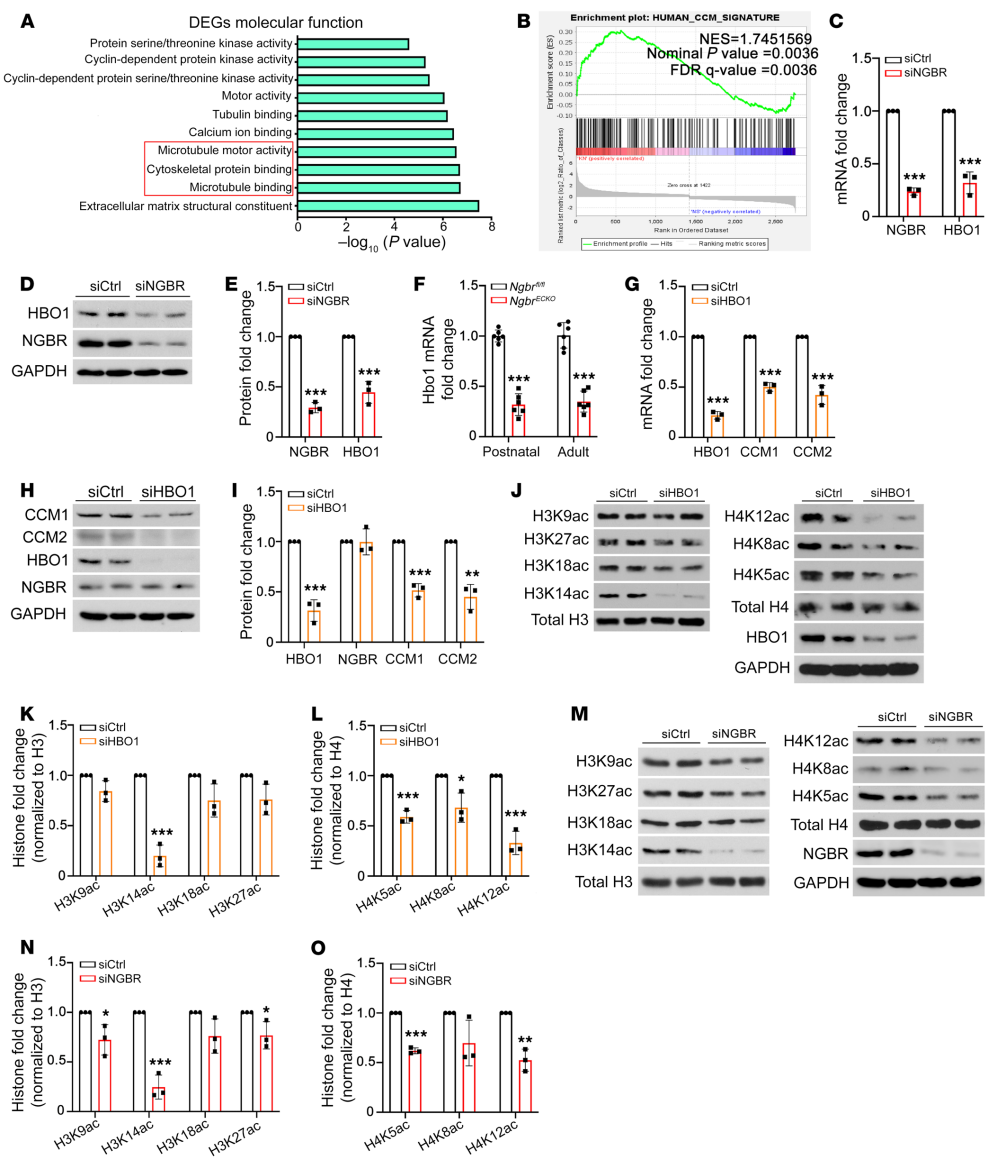
HBO1-mediated histone acetylation is involved in NGBR-regulated expression of CCM1 and CCM2 in brain ECs. (**A**) RNA-seq analysis was carried out to determine the *NGBR* deficiency–promoted transcriptomic changes in HBMVECs. GO molecular function enrichment analysis of DEGs showed enrichment in microtubule and cytoskeleton protein binding. (**B**) GSEA of RNA-seq data (siNGBR group vs. siCtrl group) showed a positive correlation with the human CCM signature gene set, with an NES equal to 1.75 and nominal *q* value and FDR *q* value both less than 0.01. (**C**–**E**) The results of RT-qPCR and Western blotting confirmed the decreased expression of HBO1 in *NGBR*-knockdown HBMVECs. (**F**) *Hbo1* mRNA level is decreased in MBMVECs isolated from the brain of postnatal and adult *Ngbr^ECKO^* mice. (**G**–**I**) *HBO1* knockdown decreased the mRNA and protein levels of CCM1 and CCM2 in HBMVECs. (**J**–**O**) Either *HBO1* or *NGBR* knockdown in HBMVECs results in a similar alteration of histone acetylation: decreased acetylation of H3K14, H4K5, and H4K12. Data are presented as mean ± SD, *n =* 3 samples per group in vitro (**C**, **E**, **G**, **I**, **K**, **L**, **N**, and **O**) and *n =* 6 mice per group in vivo (**F**). Significance was tested by 2-tailed, unpaired Student’s *t* test. *P < 0.05, **P < 0.01, ***P < 0.001.

To elucidate the underlying mechanisms by which NGBR regulates the transcription of *CCM1* and *CCM2*, we carried out RNA sequencing (RNA-seq) to examine transcriptome alterations in *NGBR*-deficient HBMVECs. The molecular function enrichment analysis of differentially expressed genes (DEGs) showed high clustering in microtubule binding, cytoskeleton protein binding, and microtubule motor activity (Figure 6A), which are closely associated with RhoA/phos-MLC signaling (48–52). Importantly, gene set enrichment analysis (GSEA) of these RNA-seq data showed a good correlation with the DEGs from human CCM lesions (53), with a normalized enrichment score (NES) of 1.75 and nominal *P* value and FDR *q* value both less than 0.01 (Figure 6B). The results of this comprehensive analysis provide a second layer of evidence to support the correlation between *NGBR* deficiency in ECs and CCM pathogenesis. However, why is *NGBR* required for preserving the expression of *CCM1* and *CCM2* in brain ECs?

We further searched the RNA-seq data for transcriptional regulators and surprisingly found the HAT *HBO1* remarkably decreased in *NGBR*-deficient HBMVECs compared with control groups. In contrast, other HATs and histone deacetylases (HDACs) were not significantly altered. The expression of genes encoding HATs (including *KAT1*, *GCN5*, *PCAF*, *CREBBP*, *EP300*, *KAT5*, *KAT6A*, *HBO1*, and *KAT8*) and HDACs (including *HDAC1–11* and sirtuins [SIRTs], including *SIRT1–7*) in *NGBR*-deficient HBMVECs was further examined by reverse transcription quantitative polymerase chain reaction (RT-qPCR) and Western blotting, respectively. The mRNA and protein expression levels showed that HBO1 is the HAT significantly decreased in *NGBR*-deficient HBMVECs (Figure 6, C–E, and Supplemental Figure 8, A–C). Moreover, *Hbo1* was also significantly decreased in MBMVECs isolated from the brain of either postnatal or adult *Ngbr^ECKO^* mice (Figure 6F). To determine the relationship between HBO1 and CCM1/2 expression, *HBO1* was knocked down in HBMVECs with validated *HBO1* siRNA. The RT-qPCR and Western blotting results demonstrated that *HBO1* knockdown resulted in downregulation of CCM1 and CCM2 expression (Figure 6, G–I). Of note, *HBO1* knockdown did not affect NGBR expression (Figure 6, H and I). As shown in Supplemental Figure 8, C and D, another HAT-encoding gene, *GCN5*, showed a slight decrease under *NGBR* knockdown. However, unlike *HBO1* (Figure 6, G–I), *GCN5* knockdown did not affect the expression of CCM1 and CCM2 (Supplemental Figure 8E). These results suggest that HBO1 may be an intermediate in the NGBR-mediated pathway to regulate the transcription of *CCM1* and *CCM2*.

As shown in our previous publication (54), NGBR has a hydrophobic cytoplasmic domain that binds to farnesylated proteins, which are involved in regulating the expression of transcription factors (55, 56). SREBP-1c is one of the NGBR-dependent transcription factors, and the *HBO1* promotor region has an SREBP-1c binding site (SRE-1), which is not present in the promotor regions of *CCM1/2/3*. As shown in Supplemental Figure 9, loss of *NGBR* downregulated SREBP-1c expression (Supplemental Figure 9, A and B), while knockdown of *SREBP-1c* using siRNA attenuated the expression of HBO1 in HBMVECs (Supplemental Figure 9, C and D). As noted herein, *SREBP-1c* knockdown did not affect the expression of NGBR (Supplemental Figure 9, C and D). Furthermore, ChIP-qPCR assay results showed that loss of *NGBR* impairs the enrichment of SREBP-1c on the promotor of the *HBO1* gene (Supplemental Figure 9, E and F). These data indicate that NGBR may regulate the transcription of *HBO1* expression through SREBP-1c.

Based on the reported HAT activity of HBO1 (33–35), we examined the effects of either *HBO1* or *NGBR* knockdown on the acetylation of H3K9/K14/K18/K27 and H4K5/K8/K12 in HBMVECs. Western blotting analysis showed that either *HBO1* or *NGBR* deficiency results in a similar alteration of histone acetylation, i.e., a significant decrease in acetylated H3K14 (H3K14ac), H4K5ac, and H4K12ac (Figure 6, J–O). Taken together, these findings indicated that *NGBR* may regulate the transcription of *CCM1* and *CCM2* through HBO1-mediated histone acetylation.

### The enrichment of HBO1-mediated H4K5 and H4K12 acetylation on the promoter regions of CCM1 and CCM2 genes.

**Figure 7:**
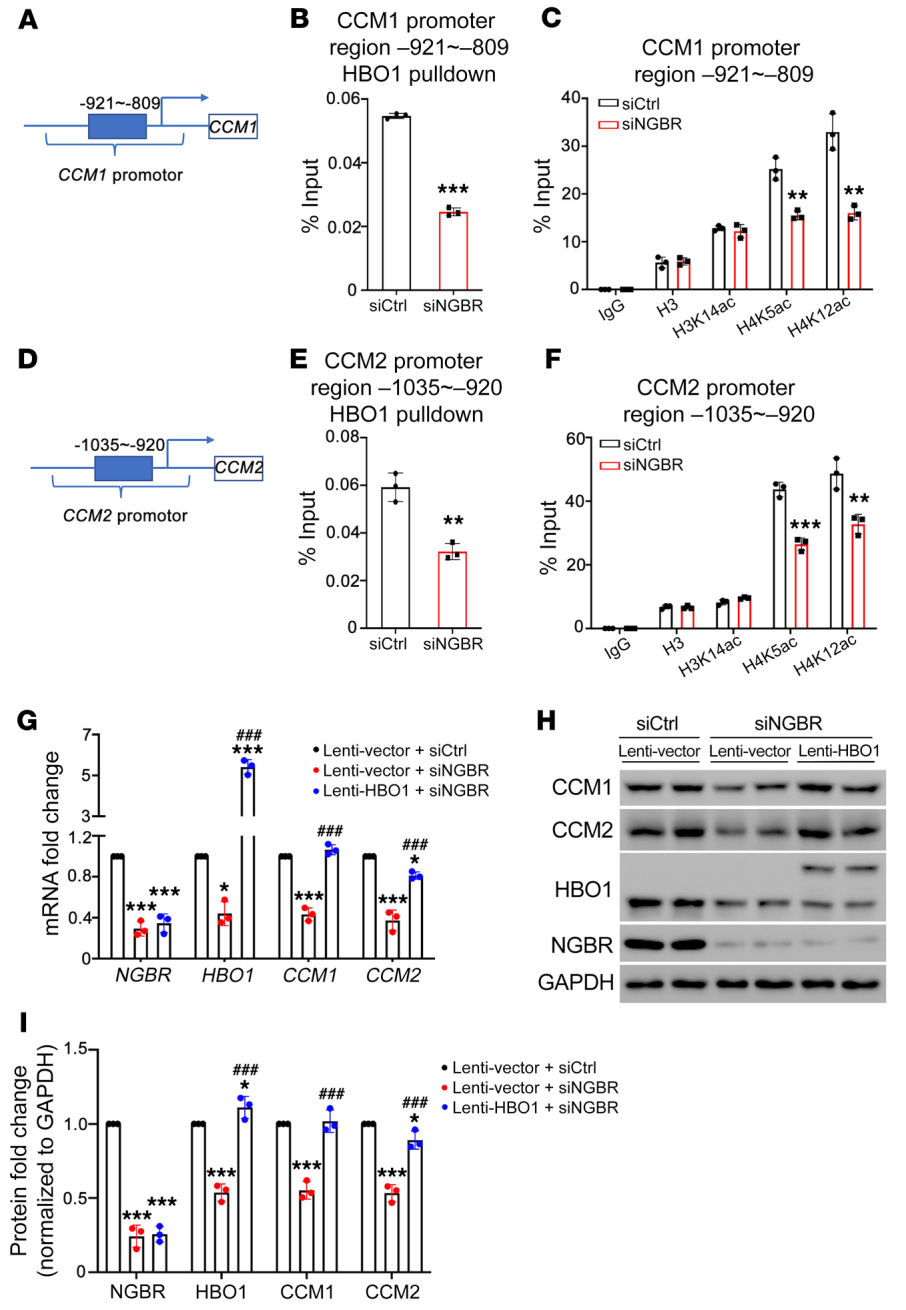
HBO1-mediated histone H4 acetylation is required for NGBR-regulated CCM1 and CCM1 expression. (**A**–**F**) ChIP-qPCR assays were performed for HBMVECs treated with either control siRNA or *NGBR* siRNA. Chromatin DNA was pulled down using respective antibodies against IgG, HBO1, H3, H3K14ac, H4K5ac, and H4K12ac. Results showed that *NGBR* knockdown ameliorated the binding of HBO1, H4K5ac, and H4K12ac on the promotor region of the *CCM1* gene (–921 to –809) (**A**–**C**) and on the promotor region of *CCM2* (–1035 to –920) (**D**–**F**). Data are presented as mean ± SD, *n =* 3 samples per group. Significance was tested by 2-tailed, unpaired Student’s *t* test (**B**, **C**, **E**, and **F**). **P < 0.01, ***P < 0.001. (**G**–**I**) Lentivirus-mediated overexpression of *HBO1* (lenti-HBO1) restored *CCM1* and *CCM2* gene transcription and protein expression in *NGBR*-deficient HBMVECs. Data are presented as mean ± SD, *n =* 3 samples per group. Significance was tested by 1-way ANOVA with Dunnett’s post hoc test. *P < 0.05, ***P < 0.001 versus siCtrl- and lenti-vector–treated group; ^###^*P* < 0.001 versus siNGBR- and lenti-vector–treated group.

It has been demonstrated that particular patterns of histone posttranslational modifications represent a code that is recognized by transcription factors via specific chromatin-binding domains (35, 57, 58). The enrichment of acetylated histone protein at the promoter region is essential for regulating gene transcription (59–61). ChIP-qPCR assay using an anti-HBO1 antibody could determine the binding of HBO1 on the promotor region of *CCM1* and *CCM2*. The results showed that HBO1 binds the region –921 to –809 of the *CCM1* promotor and the region –1035 to –920 of the *CCM2* promotor, and HBO1 binding was significantly decreased in *NGBR*-deficient HBMVECs (Figure 7, A, B, D, and E). However, the binding of HBO1 on the *CCM3* promotor region was not altered under the *NGBR*-knockdown condition (Supplemental Figure 10A). As shown in Figure 6, J–L, acetylation of H3K14, H4K5, and H4K12 is dependent on the expression of HBO1 in HBMVECs. We further carried out the ChIP-qPCR assays using anti-H3K14ac, -H4K5ac, and -H4K12ac antibodies. ChIP-qPCR results showed the enrichment of H4K5ac and H4K12ac on the promotor region of *CCM1* and *CCM2*, respectively (Figure 7, C and F). Like HBO1, the binding of H4K5ac and H4K12ac was significantly decreased in *NGBR*-deficient HBMVECs (Figure 7, B, C, E, and F). In contrast, *NGBR* knockdown in HBMVECs did not affect the binding of H3K14ac on the promotor region of *CCM1* and *CCM2*, nor did the binding of H3K14ac, H4K5ac, H4K8ac, and H4K12ac on the promotor region of *CCM3* (Supplemental Figure 10, B–E). These results indicated that NGBR regulates the transcription of *CCM1* and *CCM2* but not *CCM3* via HBO1-mediated acetylation of H4K5 and H4K12.

To further confirm whether NGBR is dependent on HBO1 in regulating the transcription of *CCM1* and *CCM2*, we carried out HBO1 rescue experiments. We first generated lentivirus carrying the *HBO1* transgene for restoring *HBO1* expression in *NGBR*-deficient HMBVECs in vitro. As we expected, *HBO1* overexpression sufficiently restored the expression of *CCM1* and *CCM2* in *NGBR*-knockdown HBMVECs (Figure 7, G–I). As noted herein, *HBO1* overexpression did not affect NGBR expression in HBMVECs. These data demonstrated that NGBR is dependent on HBO1 in regulating the expression of *CCM1* and *CCM2*.

### HBO1 overexpression restored CCM1/2 expression and vascular defects in the brain of Ngbr^ECKO^ mice.

**Figure 8:**
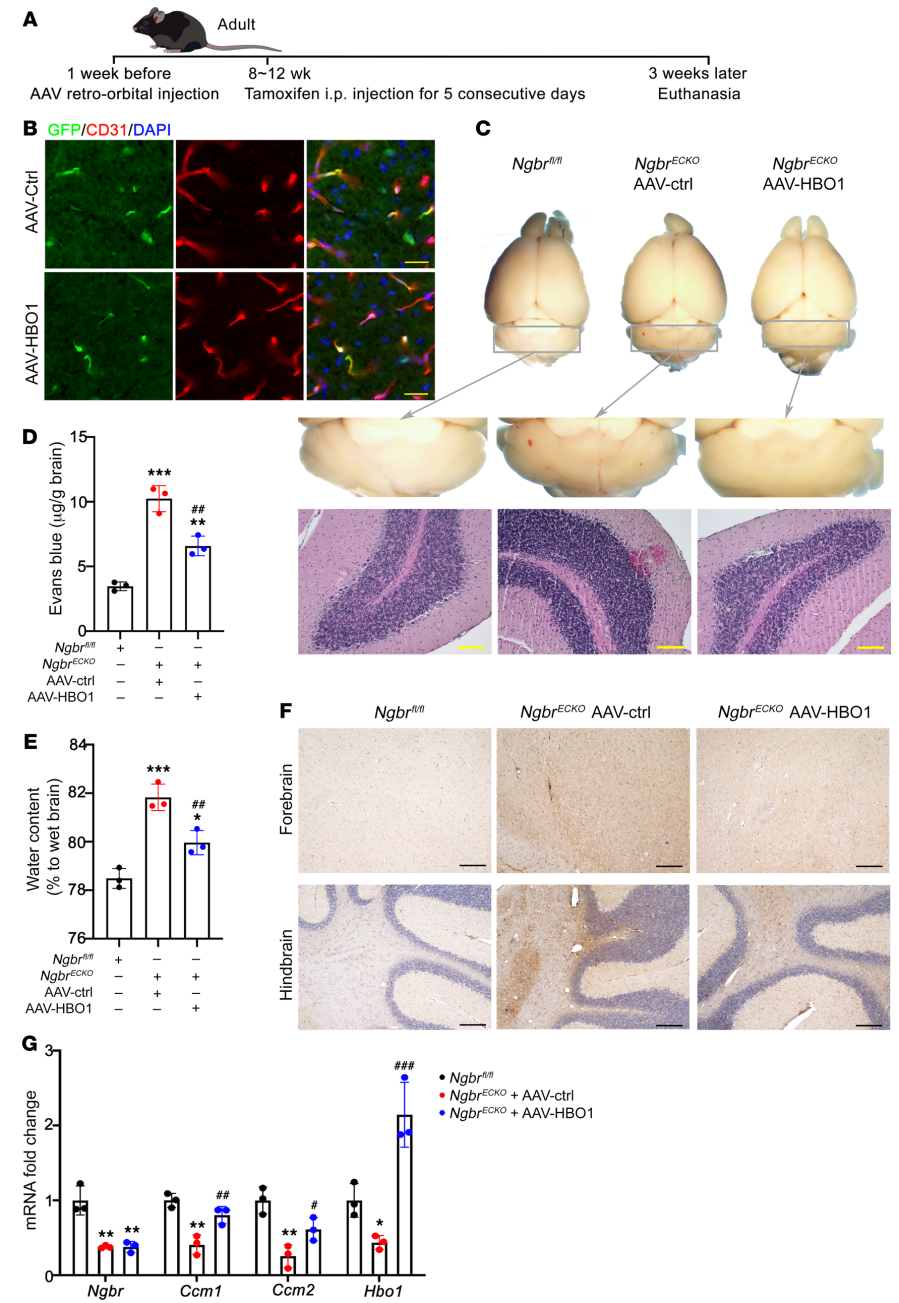
HBO1 overexpression in vivo ameliorates *Ngbr* deficiency–promoted cerebral hemorrhage and BBB leakage. HBO1 overexpression in brain ECs of *Ngbr^ECKO^* mice was achieved by the intravenous administration of AAV-BR1-GFP-HBO1 (AAV-HBO1). AAV-BR1-GFP was used as a control. (**A**) Tamoxifen was injected 1 week after AAV injection, and mice were euthanized 3 weeks after tamoxifen injection as shown in the protocol schematic. (**B**) Efficacy and localization of AAV-BR1 were determined by immunofluorescent staining of GFP. The images of GFP staining in brain sections showed HBO1-GFP expression in brain ECs (labeled by CD31, red) after tail vein injection of AAV-HBO1. Scale bars: 25 μm. (**C**) Representative whole-brain images and H&E staining showing hemorrhage sites in AAV-ctrl–injected *Ngbr^ECKO^* mice, while no obvious hemorrhage sites were observed in AAV-HBO1–injected *Ngbr^ECKO^* mice. Scale bars: 100 μm. (**D** and **E**) HBO1 overexpression significantly diminished the hyperpermeability in the brains of *Ngbr^ECKO^* mice. Permeability was determined by the quantification of Evans blue extravasation and brain water content. (**F**) The results of IgG staining showing that AAV-HBO1 injection reduced the IgG-positive staining in the brains of *Ngbr^ECKO^* mice compared with AAV-ctrl–injected *Ngbr^ECKO^* mice. Scale bars: 200 μm. (**G**) *Ccm1/2* mRNA expression was determined in MBMVECs extracted from mice. *HBO1* overexpression in vivo rescued *Ccm1* and *Ccm2* transcription in *Ngbr^ECKO^* MBMVECs. Data are presented as mean ± SD, *n =* 3 mice per group. Significance was tested by 1-way ANOVA with Dunnett’s post hoc test (**D**, **E**, and **G**). *P < 0.05, **P < 0.01, ***P < 0.001 versus *Ngbr^fl/fl^* mice treated with AAV-ctrl; ^#^*P* < 0.05, ^##^*P* < 0.01, ^###^*P* < 0.001 versus *Ngbr^ECKO^* mice treated with AAV-ctrl.

To determine the rescue effect of HBO1 in vivo, *HBO1* was overexpressed in brain ECs using AAV-BR1-HBO1-GFP (AAV-HBO1) 1 week before tamoxifen-induced deletion of *Ngbr* in ECs. The schematic protocol for animal treatment is shown in Figure 8A. The efficacy and specificity of AAV-BR1–mediated delivery of *GFP* and *HBO1* genes in ECs were determined by GFP immunofluorescence staining (Figure 8B). As shown in Figure 8B, GFP was specifically expressed in brain ECs. As visualized by the whole-mount picture and H&E staining (Figure 8C), the brain of *Ngbr^ECKO^* mice receiving AAV-HBO1 had much fewer hemorrhagic lesions compared with *Ngbr^ECKO^* mice receiving the AAV-BR1-GFP control virus. The rescue effects of *HBO1* overexpression on BBB integrity were further determined by Evans blue extravasation assay, water content, and IgG staining. Consistently, AAV-mediated *HBO1* overexpression could prevent the onset of BBB damage in the brain of *Ngbr^ECKO^* mice, as shown by the decreased permeability (Figure 8D), reduced water content (Figure 8E), and less IgG staining (Figure 8F). The RT-qPCR results further confirmed that *HBO1* overexpression restored CCM1/2 expression in MBMVECs isolated from the brains of *Ngbr^ECKO^* mice treated with AAV-HBO1 (Figure 8G). These data further demonstrated that HBO1 is essential for NGBR-dependent regulation of CCM1/2 expression in brain ECs, and *HBO1* overexpression can rescue the CCM-related lesions caused by *Ngbr* deficiency in ECs.

## Discussion

NGBR is a transmembrane protein involved in stimulating chemotaxis and morphogenesis of ECs (62). It is essential for embryonic and vascular development in mice and zebrafish (39, 41, 63). Our previous study indicated that NGBR expression decreased in the lesions of the human CCM (39). In this study, we provided critical insights into our previous findings, including comprehensive characterization of vascular lesions in the brain of *Ngbr^ECKO^* mice at both postnatal and adult stages, demonstrating CCM1 and CCM2 as critical molecules in *Ngbr* deficiency–induced endothelial dysfunction and cerebral hemorrhage, and providing what we believe is a new perspective on CCM pathogenesis in the context of HBO1-mediated histone acetylation in preserving the expression of CCM genes.

CCM is a vascular lesion that originates from ECs in the brain. Histologically identical lesions with characteristic enlarged popcorn-like capillaries are associated with all genotypes related to CCM genes (64). The lesions are isolated or clusters of enlarged leaking endothelial lumens at the capillary level, with disrupted and reduced EC junctions (8–11). Stockton et al. showed that Rho kinase hyperactivity occurs in sporadic and familial human CCM endothelium as judged by phos-MLC levels. Mutations in *CCM1* and *CCM2* genes resulting in Rho kinase hyperactivation and the Rho kinase inhibitor fasudil were found to have a rescue effect in cell culture and animal models (43). Borikova and colleagues reported a marked increase in total RhoA protein levels after the loss of expression of *CCM1*, *CCM2*, or *CCM3* and demonstrated that knockdown of RhoA effectively reverses endothelial dysfunction caused by CCM deficiency (42). McDonald et al. also presented evidence that the RhoA/phos-MLC pathway is activated in patients with sporadic CCM (65). These findings indicate that aberrant Rho-kinase activation caused by the functional loss of CCM genes are equivalent in terms of contributing to both familial and sporadic lesions. These pathological signatures of capillary-based vascular defects exhibiting enlarged and leaking endothelial lumens with hyperactivated RhoA/phos-MLC signaling and disrupted intracellular junctions are also present in the brain of *Ngbr^ECKO^* mice. GSEA comparing *NGBR*-deficient EC RNA-seq data with human CCM lesion DEGs strengthened correlations between NGBR loss and CCM pathogenesis. Rescue effects of CCM1 and CCM2 overexpression on *Ngbr* deficiency–induced endothelial defects and cerebrovascular lesions further demonstrated that CCM1 and CCM2 downregulation contributes to the pathogenesis of CCM-related vascular lesions in the brain of *Ngbr^ECKO^* mice. Our data suggest NGBR is an upstream regulator of the *CCM1* and *CCM2* genes.

Histone lysine residue acetylation, an epigenetic marker on chromatin, creates binding sites for the recruitment of transcription factors and promotes the transcription of EC genes (66–68). Histone acetylation is regulated by HATs and HDACs and is a chromatin modification involved in transcriptional activation without a change in the gene sequence (69). Arts et al. firstly reported histone H4 acetylation involved in tissue-type plasminogen activator (t-PA) gene expression in human ECs in 1995 (70). Subsequently, growing evidence has shown that acetylated histone–mediated gene expression participates in multiple endothelial functions, including endothelial barrier function. Acetylated histone H3K9 and H3K18 were demonstrated to be involved in the expression regulation of endothelial junction proteins such as claudin-5 and VE-cadherin (71, 72). Several physiopathological stimuli, including hypoxia (73), sheer stress (74, 75), reactive oxygen species (76), inflammatory cytokine (77), and high glucose (78, 79) were shown to regulate EC gene expression via altering the histone acetylation. Our recent review article provides a comprehensive update on the contributions of histone acetylation to EC functions (80). The enrichment of different histone acetylation marks at the proximal promoter regions, and even intragenic regions, corresponds to the changes in gene expression levels in ECs (81, 82).

To reveal the molecular mechanism by which NGBR regulates the transcription of *CCM1/2* genes, we firstly made an attempt to investigate all possible transcription factors in our RNA-seq data. GSEA results showed that NGBR deficiency does not result in a significant change in CCM-related transcription factors (83) in HBMVECs (Supplemental Figure 11A). Furthermore, we chose 2 transcription factors, CREB1 and ATF2, which are obviously downregulated in *NGBR*-deficient HBMVECs, and used the siRNA-mediated gene knockdown approach to determine whether and the extent to which CREB1 and ATF2 would regulate CCM1 and CCM2 expression. The knockdown of either *CREB1* or *ATF2* had no effect on CCM1 and CCM2 expression (Supplemental Figure 11, B and C). Moreover, our RNA-seq data revealed the significant suppression of *HBO1* transcription in *NGBR*-deficient HBMVECs. HBO1, a member of the MYST KAT family, is responsible for the bulk of acetylation at H4K5, H4K8, H4K12 (34), and H3K14 (35). Therefore, HBO1 is essential for embryonic development and blood vessel formation (35). As reported by Kueh et al., *Hbo1* knockout in mice was embryonic lethal, and the embryos developed enlarged blood vessels in the head region (35). Matthew et al. further demonstrated that HBO1-mediated histone acetylation regulates EC gene expression (38). In addition, Han et al. reported altered HBO1 expression in human abdominal aortic aneurysm (36), a vascular disease closely related to endothelial dysfunction (37). These pieces of evidence indicate a crucial role of HBO1 in regulating endothelial function. In this study, the results of *HBO1* knockdown and *HBO1* overexpression established the link between HBO1 and the expression of CCM1 and CCM2 in the context of *NGBR* deficiency–promoted CCM-related lesions. The contributions of HBO1-mediated histone acetylation to the transcriptional regulation of *CCM1* and *CCM2* genes were further supported by ChIP-qPCR results. These findings revealed an epigenetic regulation of CCM genes in an NGBR/HBO1-dependent manner.

Unlike genetic mutation, epigenetic disorders such as histone acetylation alterations are reversible. The tipping point of histone acetylation is maintained by HAT and HDAC enzyme families. The respective function of HATs and HDACs is to add or remove the acetyl group to or from the lysine residue, resulting in chromatin opening or closing for initiating transcription. Inhibition of the enzyme’s activity is a way to alter histone acetylation status. A wide spectrum of HDAC inhibitors is available to target different HDACs specifically. Among commonly used HDAC inhibitors, such as trichostatin A (TSA), suberoylanilide hydroxamic acid (SAHA), and nicotinamide (NAM), SAHA has been approved in clinical trials for cancer treatment (84, 85). Of note, NAM restored histone H4K12 acetylation and CCM1/2 expression in *NGBR*-deficient HBMVECs (data not shown).

As reported previously, most CCMs exhibit a 2-hit mechanism (23–25). However, somatic mutation of CCM genes is detected in only around half of sporadic CCM cases with multiple lesions (26–28), which means that the molecular mechanism responsible for CCM loss in the other half of sporadic CCM cases with multiple lesions and most of the sporadic CCM cases with a single lesion has not been elucidated. Although in recent years Knudson’s 2-hit mechanism has been well studied, besides somatic mutation, it has become clear that epigenetic alterations are additional mechanisms for gene silencing (86–88). Our previous study demonstrated that CCM1 protein expression decreased under long-term treatment with high glucose, and *Ccm1* heterozygous mice exhibit cerebral hemorrhage under streptozotocin-induced diabetes (29), which indicated that environment stimulation like high glucose might also serve as a second hit. The mechanisms of sporadic CCM are complicated and still under investigation. More new genes have been found to promote the pathogenesis of sporadic CCM, and even “3-hit mechanisms” have recently been proposed (89). Because there are many unknown contributors, the strategy to upregulate CCM genes might not work for all cases of sporadic CCM, especially those with CCM gene mutation or those with CCM downstream gene mutation, such as gain-of-function *MEKK3* (*MAP3K3*) mutations (90, 91). Our findings of histone acetylation–mediated regulation of *CCM1* and *CCM2* genes provide a perspective on the epigenetic contributions to certain types of sporadic CCM pathogenesis, indicating epigenetic interventions may be an alternative approach for preventing the onset of CCM disease. However, our current studies only focus on the early-stage lesions such as CCM1/2 deficiency–promoting EC junction defects and enlarged leaking cerebrovascular lesions. We may need brain EC–specific *Ngbr*-knockout mice to reveal the late-stage lesions in our future investigations. Our findings also still need further investigation to support their clinical relevance. Meanwhile, our rescue experiments only provide a proof of principle for the contributions of CCM1/2 and HBO1 deficiency to the pathogenesis of cerebrovascular malformations in the brain of *Ngbr^ECKO^* mice. Further investigations are needed to support the therapeutic implications.

In summary, our study demonstrated that NGBR- and HBO1-mediated histone acetylation is required for preserving the expression of CCM1 and CCM2 in ECs. Downregulation of CCM1 and CCM2 contributes to the vascular lesions occurring in the brains of *Ngbr^ECKO^* mice. Our data provide an insight into the underlying mechanism by which histone acetylation regulates the transcription of *CCM1* and *CCM2* genes. Our findings suggest that NGBR- and HBO1-mediated histone H4 acetylation may be a potential target for preventing the onset and progression of sporadic CCM promoted by CCM1 and CCM2 deficiency.

## Methods

### Animals

*Cdh5*-Cre^ERT2^*Ngbr^fl/fl^* mice were generated as previously reported (39). To knock out *Ngbr* in ECs in vivo, neonatal mice from mated *Cdh5*-Cre^ERT2^ *Ngbr^fl/fl^* and *Ngbr^ECKO^* adult mice were injected with tamoxifen (T5648, Sigma-Aldrich). Fresh tamoxifen solution (20 mg/mL) was prepared in sterile corn oil. Preliminary results showed that postnatal *Ngbr^ECKO^* mice started to show symptoms like seizure and ataxia as well as exhibit vessel dilation histologically starting on P6, and most of them died after P12. Adult mice did not show any behavioral symptoms, but they also encountered survival issues nearly 4 weeks after inducible depletion of *Ngbr*. We checked adult mouse brains at 2, 3, and 4 weeks after tamoxifen injection. Vessel lesions were successfully induced at 3 weeks after tamoxifen injection and most of the mice survived until 4 weeks after inducible depletion. So, we chose P7–P12 as the timing of analyses for the postnatal model and 3 weeks after tamoxifen injection as the timing of analyses for adult mice. Neonatal mice from mated *Cdh5*-Cre^ERT2^ *Ngbr^fl/fl^* females and males were injected with tamoxifen (50 μg per mouse) for 3 consecutive days after P1, and genotype was determined when euthanized (P7–P12). Alternatively, *Ngbr^ECKO^* adult mice at 8–12 weeks were injected with tamoxifen (2 mg per mouse) for 5 consecutive days and euthanized after 3 weeks. In some experiments, adult mice were injected via tail vein with AAV-BR1 expressing *CCM1* (2.5 × 10^11^ gc/mouse), *CCM2* (1.8 × 10^11^ gc/mouse), *HBO1* (2.5 × 10^11^ gc/mouse), or AAV-ctrl 1 week before tamoxifen injection. Postnatal mice were genotyped after euthanasia, and adult mice were ear tagged. We performed blinded analyses for the experiments afterward. We did not exclude animals except for unexpected death due to the intrinsic lethality issues.

### Constructs, lentiviruses, and AAV

Lentivirus expressing *CCM1* (NM_194456.1), *CCM2* (NM_001029835.2), *HBO1* (NM_007067.5), and control lentivirus harboring no transgene was generated using the second-generation lentivirus packaging system with packaging plasmid psPAX2 (12260, Addgene) and envelope plasmid pVSV-G (12259, Addgene). *CCM1*, *CCM2*, and *HBO1* transgene plasmids were in the pWPXLD (12258, Addgene) background. All constructs were confirmed by DNA sequencing. AAV-BR1 vectors harboring *CCM1*, *CCM2*, and *HBO1*, and control AAV-BR1 were obtained from Vector Laboratories.

### Evans blue extravasation and FITC-dextran perfusion

Neonatal and adult *Ngbr^fl/fl^* or *Ngbr^ECKO^* mice with or without AAV-BR1 injection were weighed and anesthetized with isoflurane. Evans Blue (E2129, Sigma-Aldrich) solution (2% in sterile PBS; 10 μL/g body weight) was injected into the retro-orbital plexus of neonatal mice or the tail vein of adult mice. Two hours (for neonatal mice) or 4 hours (for adult mice) after injection, mice were euthanized and transcardially perfused with PBS. To determine water content, brain tissues were harvested, weighed, and dried at 60°C for 48 hours and weighed again. Evans blue was extracted using 1 mL formamide at 55°C for 16 hours and determined by absorbance at 630 nm with a spectrophotometer. FITC-dextran (50 mg/mL in sterile PBS; FD2000S, Sigma-Aldrich) solution was prepared, protected from light, and then injected (10 μL/g body weight) into the retro-orbital plexus of neonatal mice or the tail vein of adult mice. At 5 minutes after injection, mice were euthanized, and brains were harvested and fixed for sectional fluorescent immunostaining.

### Histologic examination

Mouse brains were harvested, fixed with 4% paraformaldehyde (PFA) overnight, embedded in OCT or paraffin, and sectioned (8 μm or 30 μm thickness). Cells under different treatments were cultured on chamber slides (Nunc Lab-Tek II Chamber Slide System, 154534, Thermo Fisher Scientific). H&E staining, IgG staining, and immunofluorescence staining were performed on either brain tissues or cells.

#### H&E staining.

Paraffin-embedded mouse brain sections were dewaxed and rehydrated and then stained in 0.1% hematoxylin (MHS16, Sigma-Aldrich) for 20 minutes after being rinsed with tap water for 10 minutes. Slides were washed with distilled water and 95% alcohol, and then stained with 0.5% alcoholic eosin Y (1024390500, Sigma-Aldrich) for 30 seconds. After gradual dehydration, slides were mounted and photographed.

#### IgG staining.

Paraffin-embedded mouse brain sections were dewaxed, rehydrated, and then incubated with biotinylated anti–mouse IgG antibody (1:200; Vector Laboratories) for 1 hour at room temperature. Slides were then washed for 5 minutes in PBS and incubated with prepared VECTASTAIN Elite ABC Reagent (Vector Laboratories). After another 5-minute wash in PBS, slides were incubated in a peroxidase substrate solution for stain development and rinsed in tap water. Slides were then counterstained with hematoxylin, cleared, mounted, and photographed.

#### Immunofluorescence staining.

OCT-embedded mouse brain sections were washed in PBS twice and then blocked and permeabilized in 5% donkey serum in PBS with 0.3% Triton X-100 for 1 hour at room temperature. After blocking and permeabilization, slides were incubated with primary antibodies, as described in Supplemental Table 1, overnight at 4°C, and then washed with PBS 3 times and incubated with proper secondary antibodies at room temperature for 2 hours. After washing in PBS 3 more times, slides were stained with DAPI (0.5 μg/mL) for 10 minutes and mounted with an anti-fade mounting medium (P36970, Invitrogen), and then photographed. Primary HBMVECs (ACBRI 376, Cell Systems) were fixed with 4% PFA for 15 minutes, washed with PBS, permeabilized with 0.1% Triton X-100 in PBS for 5 minutes, and then blocked with blocking buffer for 30 minutes. Primary and secondary antibody application, DAPI staining, mounting, and photographing methods were the same as for tissue staining.

### Electron microscopy

Brain tissue samples were fixed in 4% glutaraldehyde in 0.1 M sodium cacodylate buffer, pH 7.5, washed in sodium cacodylate buffer, postfixed in buffered 1% osmium tetroxide, stained en bloc with a saturated solution of uranyl acetate in 40% ethanol, dehydrated in a graded series of ethanol, infiltrated in propylene oxide with Epon epoxy resin (LADD LX112, Ladd industries), and embedded. The blocks were sectioned with a Reichert Ultracut microtome at 70 nm. The resulting grids were then poststained with 1% aqueous uranyl acetate followed by 0.5% aqueous lead citrate and analyzed on a Zeiss EM 900 transmission electron microscope retrofitted with an SIA L3C digital camera.

### MBMVEC extraction

MBMVECs were extracted from *Ngbr^fl/fl^* and *Ngbr^ECKO^* mice with or without AAV-BR1 injection as previously described (92, 93). Briefly, mouse brains were collected and minced into small pieces after careful removal of the brainstem, surface vessels, and leptomeninges. Tissues were then digested with 1 mg/mL collagenase II (17101015, Thermo Fisher Scientific) and 10 μg/mL DNase I (10104159001, Sigma-Aldrich) at 37°C for 2 hours. After centrifugation at 500*g* for 5 minutes at 4°C, the lower layer was resuspended in 17% Percoll (P1644, Sigma-Aldrich) and centrifuged at 1000*g* for 15 minutes at 4°C. The precipitate was collected, digested with 1 mg/mL collagenase (Dispase, SCR139, Sigma-Aldrich) and 0.01 mg/mL DNase I at 37°C for 1 hour and then centrifuged at 500*g* for 5 minutes at 4°C. The precipitate was resuspended in a Percoll gradient and centrifuged at 1000*g* for 15 minutes at 4°C. Cells suspended at the interphase were harvested, centrifuged at 500*g* for 5 minutes at 4°C, and then lysed for Western blotting or mRNA isolation.

### EC monolayer permeability assay

The integrity of the HBMVEC monolayer was assessed by the penetration rate of FITC-dextran (FD40S, Sigma-Aldrich) through monolayer cells following a previous report (94). Briefly, HBMVECs in different groups were seeded confluently on Transwell inserts (24-well format; 0.4 mm pore; 3467, Corning). Fresh culture medium (150 mL) with 100 μg/mL FITC-dextran was added into the upper chamber, and 1 mL fresh culture medium without FITC-dextran was added to the lower chamber. Samples (50 μL) were retrieved from the lower chamber at 0, 30, and 60 minutes and the medium replaced with fresh culture medium. Samples were then analyzed by a fluorescence microplate reader (PerkinElmer) with a wavelength setting of 488/510 nm (ex/em). The permeability coefficient (cm/min) was determined using *V*/(*SA* × *Cd*) × (*Cr*/*T*), where *V* is the medium volume in the receiver chamber, *SA* is the surface area of the cell monolayer, *Cd* is the concentration of FITC-dextran in the donor chamber at time 0, and *Cr* is the concentration of FITC-dextran in the receiver chamber at sampling time *T*. Permeability changes are presented as percentage of control.

### Western blotting analysis

Proteins extracted either from HBMVECs or isolated MBMVECs from *Ngbr^fl/fl^* and *Ngbr^ECKO^* mice were resolved using 8%–14% SDS-PAGE at 20–30 μg/lane, transferred to nitrocellulose membranes (RPN303D, GE Healthcare), and blocked for 1 hour in Tris-buffered saline (TBS) containing 0.5% Tween 20 and 5% nonfat milk at room temperature. The membranes were then incubated with primary antibodies (Supplemental Table 1) overnight at 4°C. After washing 3 times in TBS/0.5% Tween 20, membranes were incubated with corresponding HRP-conjugated secondary antibodies for 2 hours at room temperature. Immunoreactivity was detected by chemiluminescence. See complete unedited blots in the supplemental material.

### RT-qPCR

RNA was isolated from either HBMVECs or MBMVECs using TRIzol (15596018, Thermo Fisher Scientific). Extracted total mRNA was then reverse transcribed using iScript Reverse Transcription Supermix (1705541, Bio-Rad). qPCR was then performed using iTaq Universal SYBR Green Supermix (1725121, Bio-Rad). Primers used are shown in Supplemental Table 2. The mRNA expression level of GAPDH was set as housekeeping control.

### RNA-seq

Total RNA was isolated from HBMVECs using an RNeasy Mini Kit (74106, Qiagen) and quality was assessed by fragment analysis (Agilent). The qualified RNA of HBMVECs transfected with either control siRNA or NGBR siRNA was sent to the Genomic Science and Precision Medicine Center (GSPMC) at the Medical College of Wisconsin for RNA-seq on an Illumina HiSeq. Sequencing reads were processed through the MAPR-Seq workflow (https://bioinformaticstools.mayo.edu/research/maprseq/) with differential expression analysis completed with EdgeR software (http://www.bioconductor.org/packages/release/bioc/html/edgeR.html). Data were deposited in the NCBI’s Gene Expression Omnibus database (GEO GSE198351).

### ChIP-qPCR assay

HBMVECs under different treatments were processed for ChIP assay using SimpleChIP Plus Kits (9004 and 9005, Cell Signaling Technology) according to the manufacturer’s instructions. Antibodies used for ChIP assay are described in Supplemental Table 1. DNA samples obtained after immunoprecipitation were analyzed by qPCR. Gene promotor–specific primers are listed in Supplemental Table 2. Anti–histone H3 antibody and anti-IgG antibody were used as positive control and negative control, respectively.

### Gene Ontology molecular function enrichment analysis

Genes with log(fold change) (logFC) greater than 1 or less than –1 and *P* value less than 0.05 from transcriptome RNA-seq data (Supplemental Table 3) obtained from HBMVECs were analyzed using the ToppGene webtool (https://toppgene.cchmc.org). Gene Ontology (GO) molecular function enrichment analysis was performed.

### GSEA

DEGs from human CCM lesions were obtained from a published report (53). Those DEGs were defined as a “human CCM signature” gene set. The 12,284 genes detected in HBMVECs (control siRNA vs. NGBR siRNA) were ranked using GSEA based on a signal-to-noise ratio ranking metric. Preranked GSEA was performed using the human CCM signature–associated genes for their association with human CCM disease expression pattern in the HBMVEC groups described above. GSEA was conducted using MSigDB v7.3 (https://www.gsea-msigdb.org/gsea/msigdb/). A gene set was considered significantly enriched when the FDR was less than 0.25.

### Statistics

Quantification of immunofluorescence images was conducted with ImageJ software (NIH). Statistical analysis was performed using GraphPad Prism and SPSS software. Data are presented as mean ± SD. Statistical testing between 2 groups was performed using a 2-tailed, unpaired Student’s *t* test. Multiple comparisons among groups of more than 2 were performed using 1-way ANOVA and Dunnett’s post hoc test. Differences were considered statistically significant when *P* was less than 0.05.

### Study approval

All animal studies were approved by the Institutional Animal Care Use Committees of the Medical College of Wisconsin and New York University Langone Health.

## Author contributions

ZF, WH, XS, XW, and JM performed and interpreted the majority of the experiments. UR carried out retina staining, and BM carried out transcription factor GSEA. TP and LR carried out electron microscopy experiments. QRM, ZF, and WH conceived the project, designed the experiments, and wrote the manuscript. QRM supervised the project. All the authors commented on the manuscript.

## Supplementary Material

Supplemental data

Supplemental table 3

Supplemental video 1

Supplemental video 2

## Version 1 03/22/2022

In-Press Preview

## Version 2 05/02/2022

Electronic publication
