# Supplementary material for: NOGOB receptor deficiency increases cerebrovascular permeability and hemorrhage via impairing histone acetylation–mediated CCM1/2 expression
Source: J Clin Invest. 2022 May 2;132(9):e151382. doi: 10.1172/JCI151382 (PMC9057619; doi:10.1172/JCI151382)

Supplementary figures of manuscript 15138-JCI-RG-RV-2

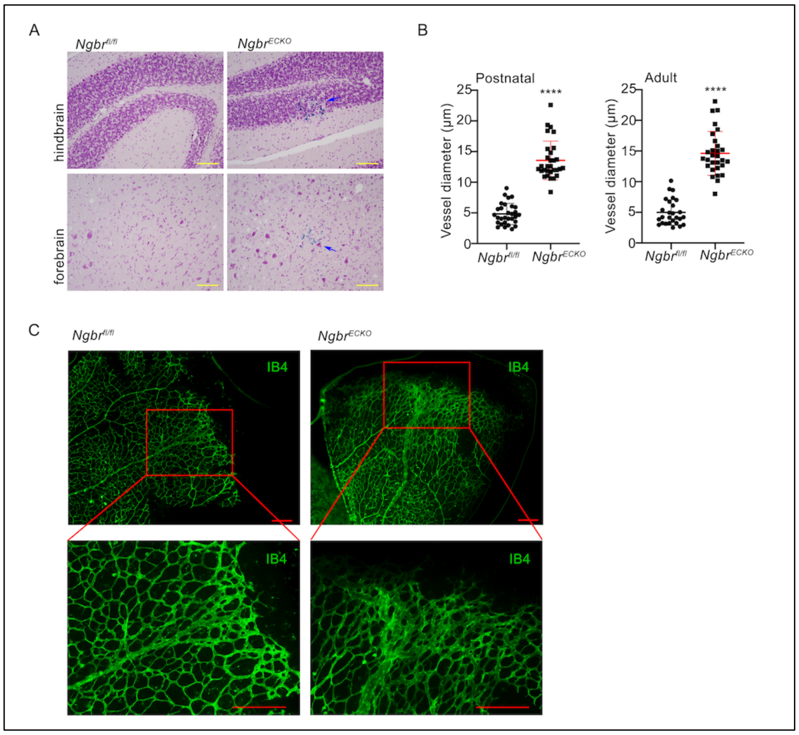

Figure S1 A. Hemosiderin staining using Iron Stain Kit indicated stale hemorrhage in both forebrain and hindbrain of adult mice. Arrows pointed to the blue-stained positive sites. Scale bar: 50 μm. B. Microvessel diameter was calculated from IB4 staining images of *Ngbr*$^{fl/fl}$ and *Ngbr*^{ECKO} mice. Results showed microvessel diameter significantly increased in *Ngbr*^{ECKO} mice. Images of lesion sites from *Ngbr*^{ECKO} group and similar sites from *Ngbr*$^{fl/fl}$ group were taken. Microvessels in 6 images randomly selected from 3 mice per group were measured. Data are presented as mean ± SD. Significance was tested by 2-tailed unpaired Student's t-test. *****P*<0.0001. C. Retina were extracted from postnatal (P7) mice of both *Ngbr*$^{fl/fl}$ and *Ngbr*^{ECKO} groups. ECs were stained with IB4 (green). Results showed that venous vessels in the retina of *Ngbr*^{ECKO} group were enlarged. Scale bar: 200 μm.

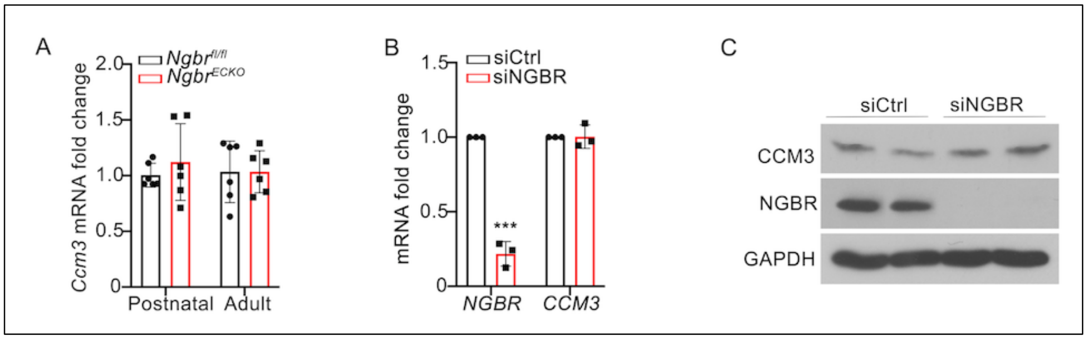

Figure S2 A. Ccm3 mRNA levels were detected in MBMVECs isolated from *Ngbr*$^{fl/fl}$ and *Ngbr*^{ECKO} groups at both postnatal and adult stages. Results showed that the *Ccm3* mRNA level didn't change in *Ngbr*^{ECKO} MBMVECs. n=6 mice per group. B-C. RT-qPCR and western blot showed no significant change of *CCM3* expression in *NBGR* deficient HBMVECs. Data are presented as mean ± SD. Significance was tested by 2-tailed unpaired student's t-test. ****P*<0.001, n=3 samples per group.

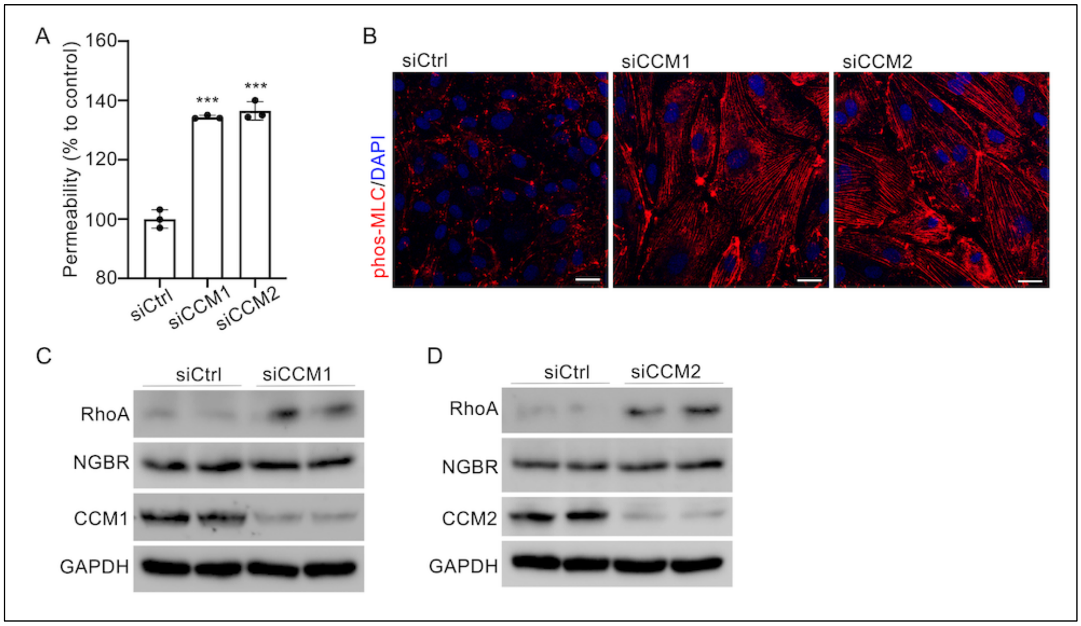

Figure S3 A. Knockdown of either *CCM1* or *CCM2* significantly increases endothelial permeability. Data are presented as mean ± SD. Significance was tested by 2-tailed unpaired student's t-test. ****P*<0.001 *vs.* siCtrl group, n=3 samples per group. B-D. Immunofluorescent staining and western blot showed that knockdown of either *CCM1* or *CCM2* increases RhoA expression and phos-MLC signaling. Phos-MLC and RhoA expression was much higher in *CCM1* or *CCM2* knockdown group compared to siCtrl group. Scale bar: 20 μm.

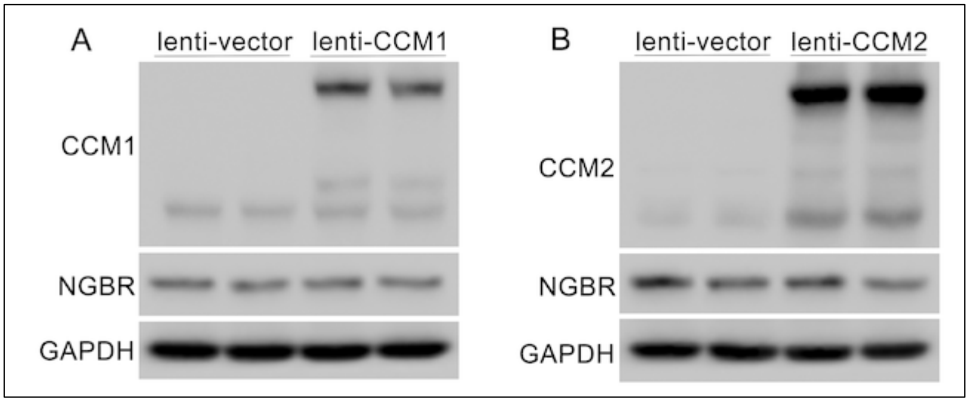

Figure S4 A-B. Western blot showed that lentivirus transfection efficiency was sufficient. CCM1 and CCM2 were overexpressed in HBMVECs when transfected with lentivirus harboring *CCM1* or *CCM2* gene. As noted herein, NGBR protein levels had no change.

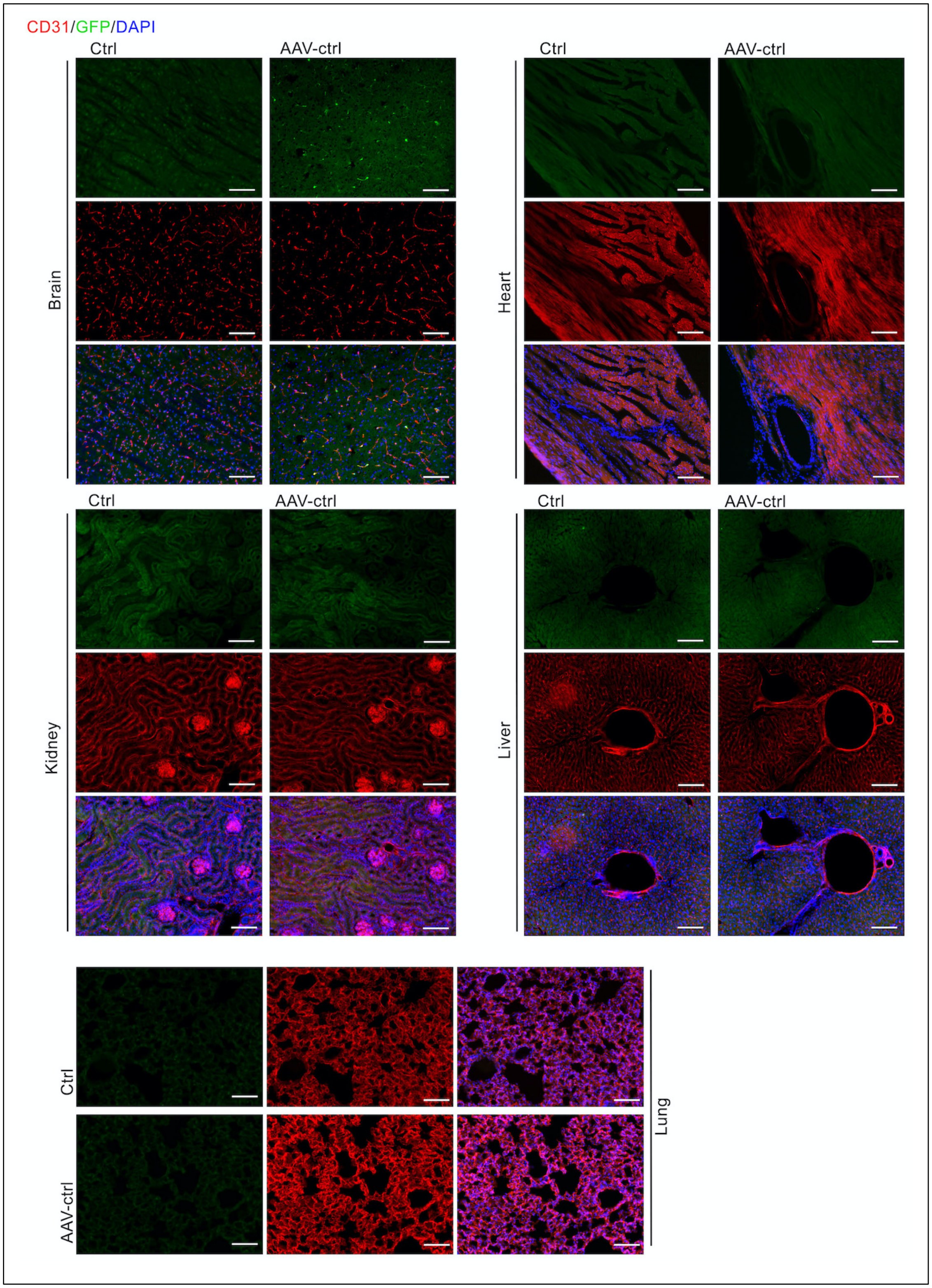

Figure S5 Mice were injected with AAV-BR1-GFP (AAV-ctrl) and vehicle (Ctrl) via tail vein. Mice were euthanized and perfused by PBS two weeks later. Organs were obtained, fixed, embedded, sectioned, and immunofluorescent stained with CD31, GFP, and DAPI. Results showed that positive GFP staining presented in brain ECs but not in any ECs in the heart, kidney, liver, and lung. Scale bar: 50 μm.

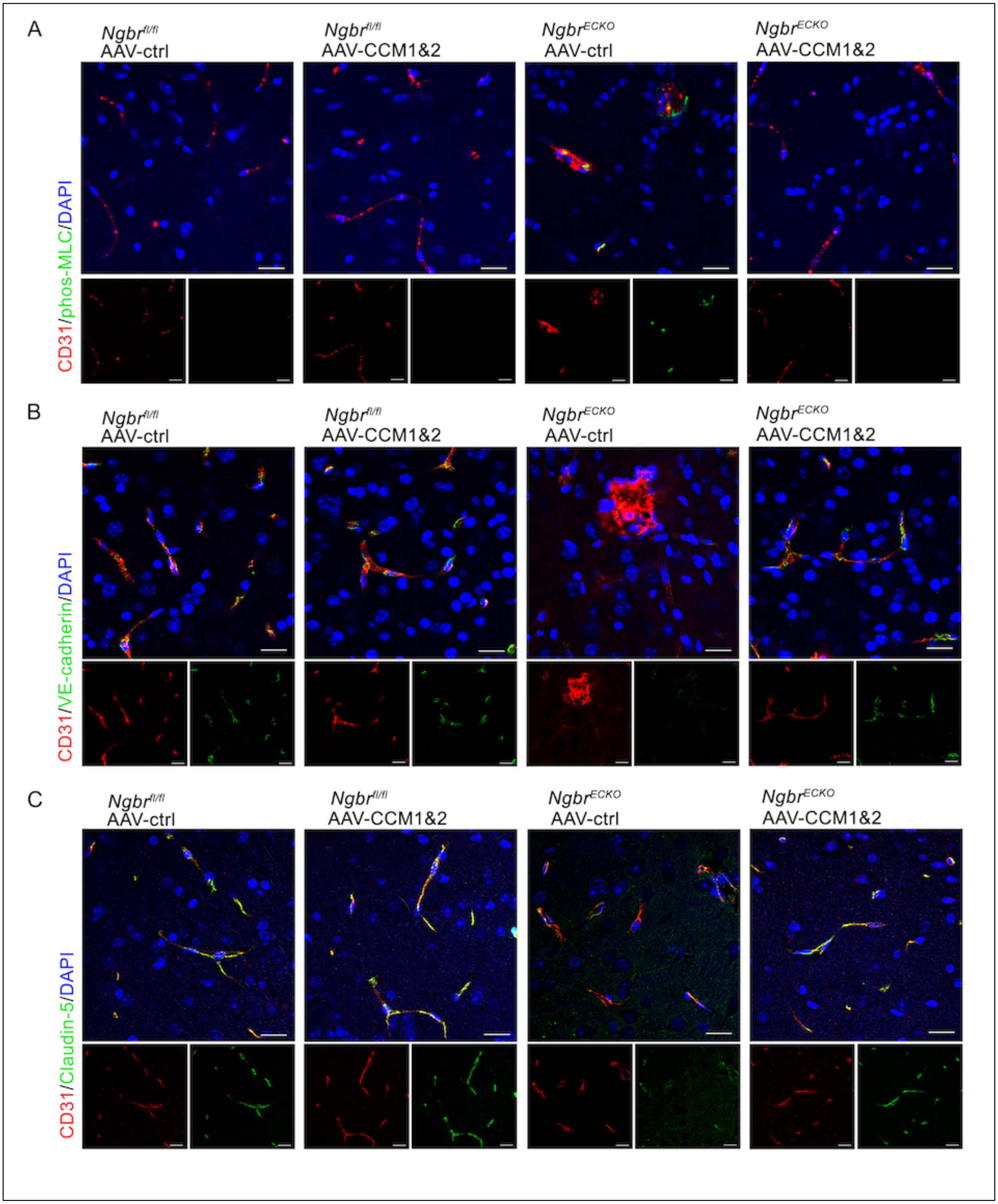

Figure S6 A-C. Immunofluorescent staining showed the reduction of phos-MLC (A), as well as restoration of AJs (VE-cadherin, B) and TJs (Claudin-5, C) protein levels in the brain ECs of *Ngbr*^{ECKO} mice overexpressing *CCM1* and *CCM2*. Scale bar: 20 μm.

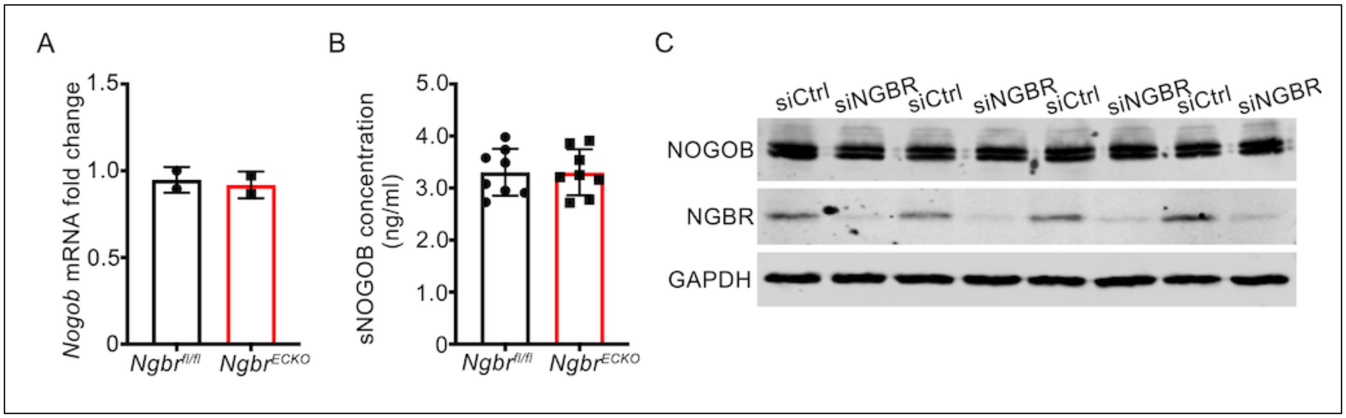

Figure S7 A. *Nogob* mRNA level did not change in *Ngbr* deficient MBMVECs extracted from *Ngbr*^{ECKO} mice. Each point represented an MBMVECs sample from 4 mouse brains. B. The concentration of sNOGOB was detected by ELISA. The levels of NOGOB in serum from *Ngbr*^{ECKO} mice are the same as that of *Ngbr*$^{fl/fl}$ mice. Data are presented as mean ± SD. Significance was tested by 2-tailed unpaired student's t-test. No significant differences were detected. C. NOGOB expression in HBMVECs was detected by western blot. NOGOB expression remained the same under *NGBR* siRNA treatment.

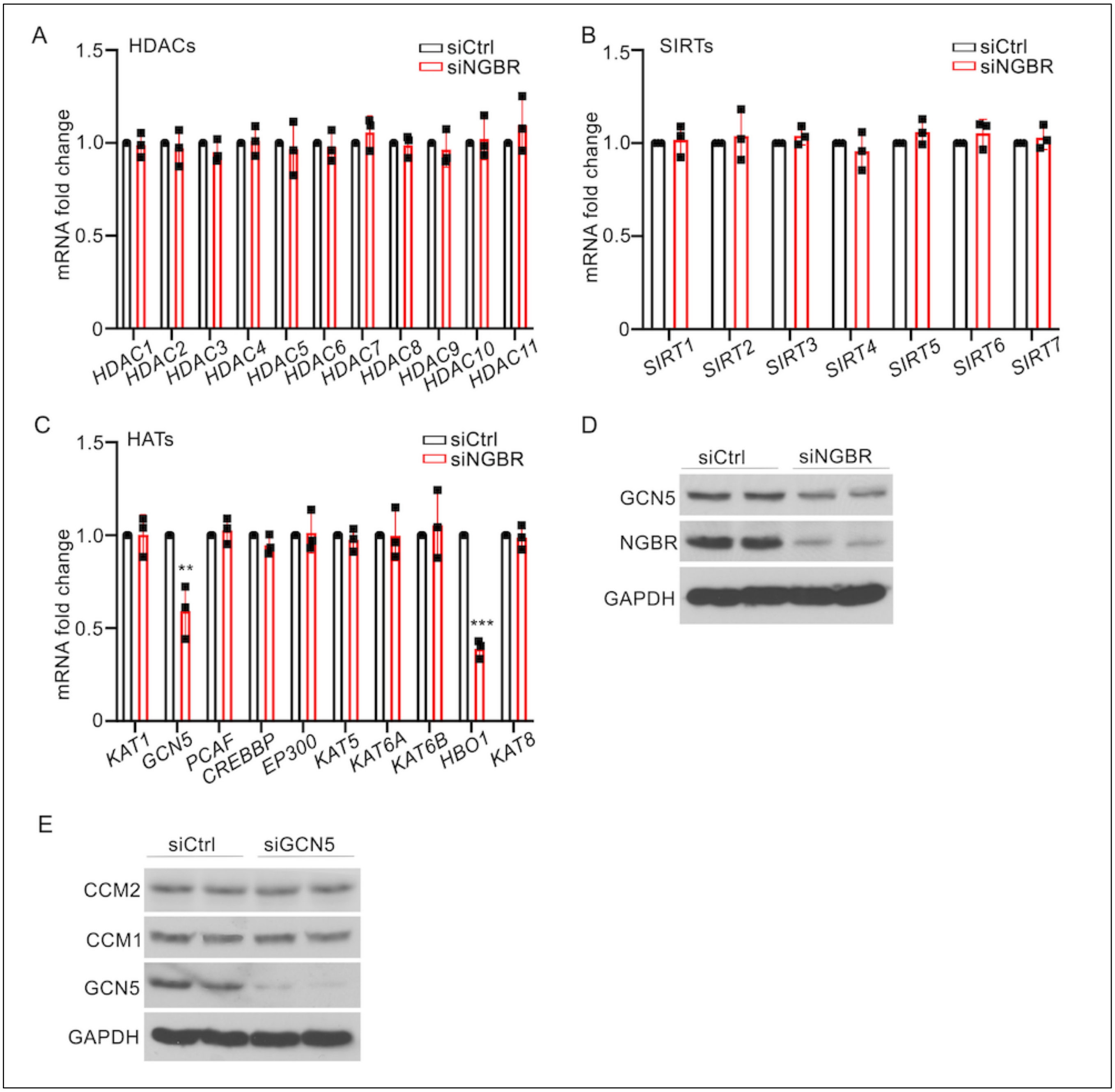

Figure S8 A-C. HDACs, SIRTs, and HATs expression in RNA-seq data (siNGBR group *vs.* siCtrl group) were confirmed by RT-qPCR. The results showed that the mRNA level of *HBO1* decreased dramatically, and the mRNA level of *GCN5* also decreased. Data are presented as mean ± SD. Significance was tested by 2-tailed unpaired student's t-test. ***P*<0.01, ****P*<0.001. n=3 samples per group. D-E. Western blot results showed GCN5 decreased in *NGBR* deficient HBMVECs while *GCN5* siRNA treatment didn't show any influence on CCM1 and CCM2 expression in HBMVECs even though the knockdown efficiency was sufficient.

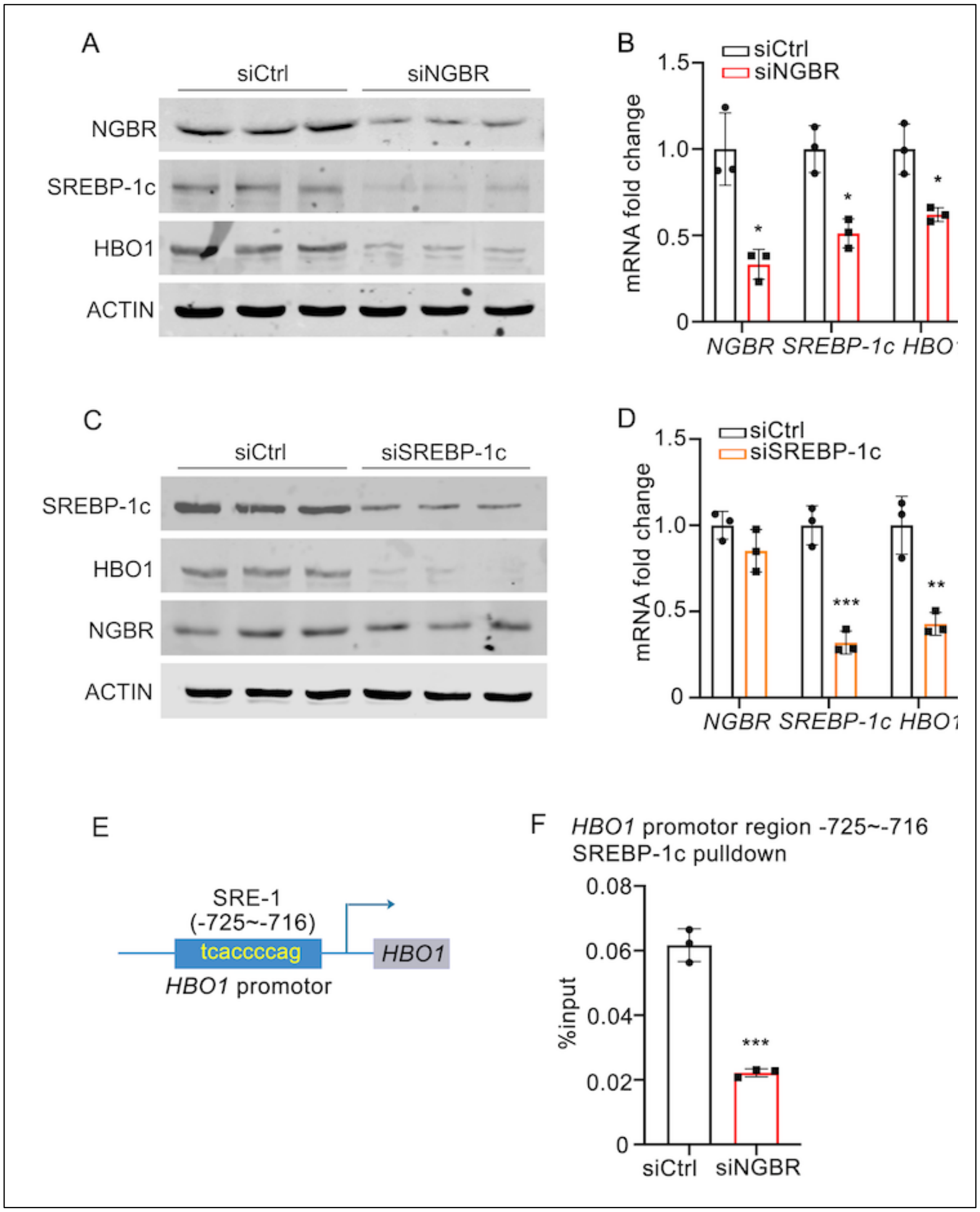

Figure S9 A-B. Both protein and mRNA levels of SREBP-1c and HBO1 were decreased in *NGBR* knockdown HBMVECs. Data are presented as mean ± SD. Significance was tested by 2-tailed unpaired student's t-test. **P*<0.05, n=3 samples per group. C-D. HBO1 protein and mRNA levels were reduced in *SREBP-1c* knockdown HBMVECs. As noted herein, NGBR expression did not change. Data are presented as mean ± SD. Significance was tested by 2-tailed unpaired student's t-test. ***P*<0.01, ****P*<0.001, n=3 samples per group. E. The binding site of SREBP-1c (SRE-1) on the *HBO1* gene promotor region. F. ChIP-qPCR assays were performed using HBMVECs treated with either control siRNA or *NGBR* siRNA. Chromatin DNA was pulled down using an antibody of SREBP-1c. Results showed that the binding of SREBP-1c on the *HBO1* promotor region (-725~-716) was significantly decreased in NGBR deficient HBMVECs. Data are presented as mean ± SD. Significance was tested by 2-tailed unpaired student's t-test. ****P*<0.001, n=3 samples per group.

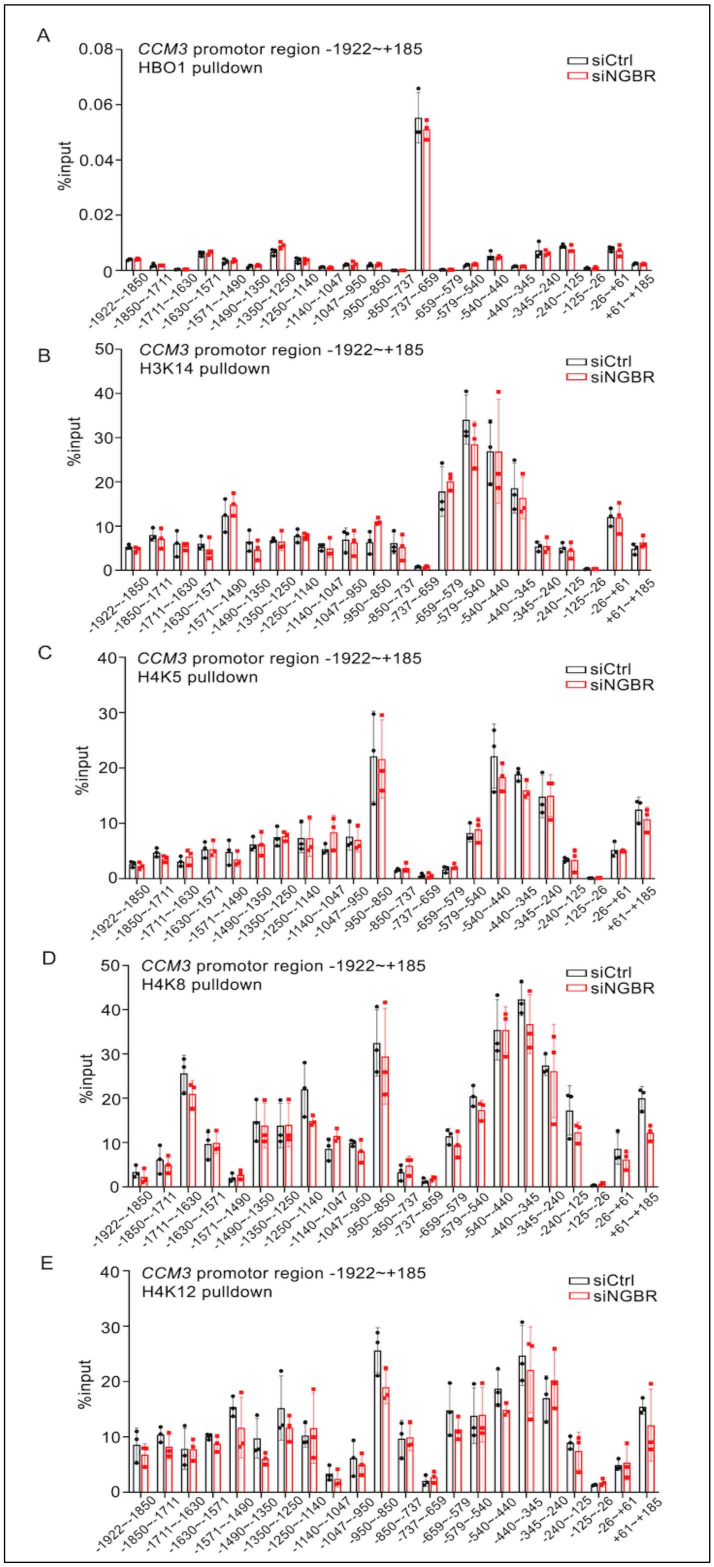

Figure S10 A-E. ChIP-qPCR assays were performed using HBMVECs treated with either control siRNA or *NGBR* siRNA. Chromatin DNA was pulled down using antibodies of HBO1, acetylated H3K14, H4K5, H4K8, and H4K12. Chromatin DNA pulled down using IgG and H3 was performed as the negative and positive control (data not shown). Results showed that *NGBR* knockdown did not affect the binding of HBO1, acetylated H3K14, and acetylated H4K5/K8/K12 on the promotor region of the *CCM3* gene. Data are presented as mean ± SD. Significance was tested by 2-tailed unpaired student's t-test.

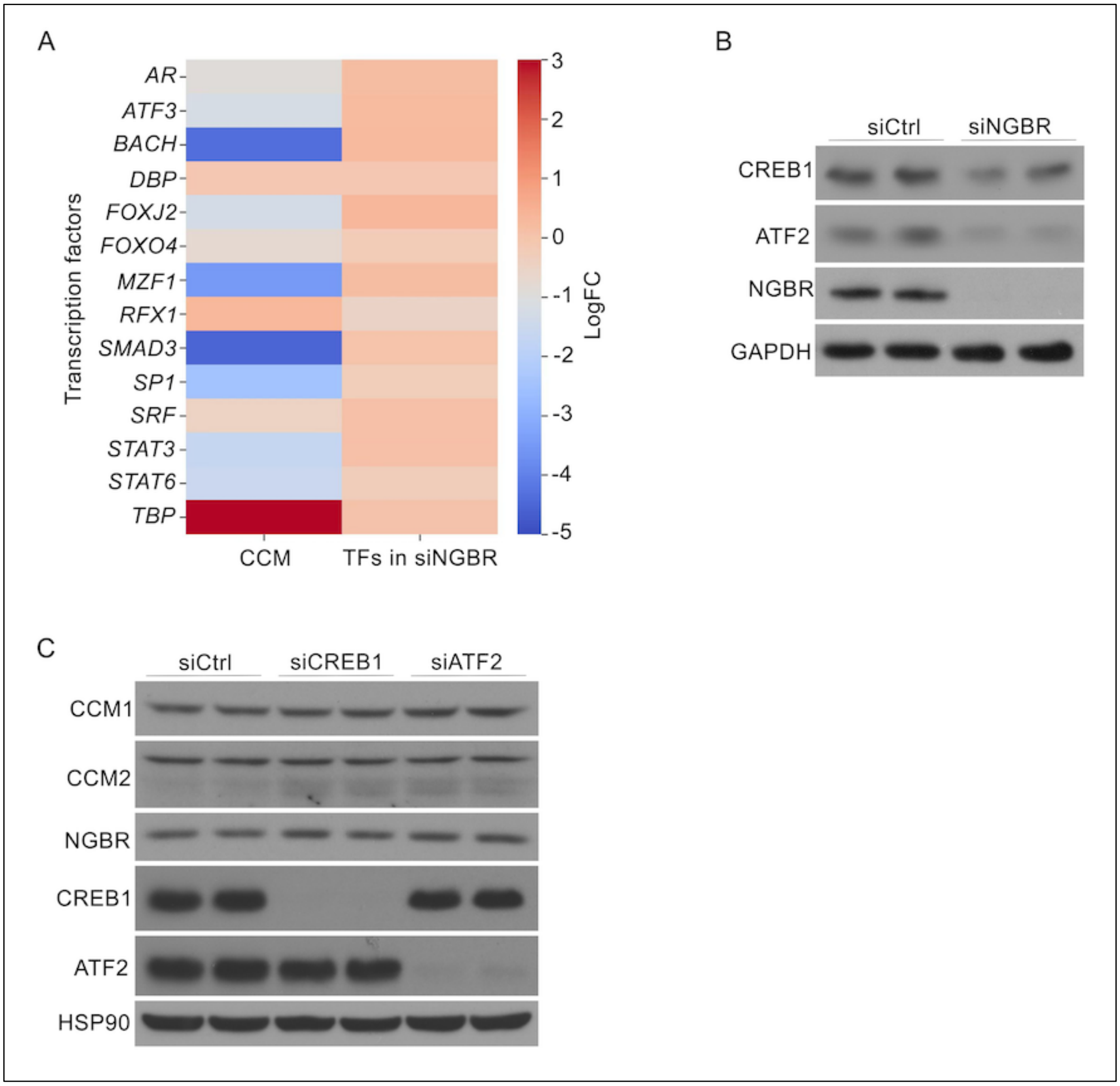

Figure S11 A. GSEA analysis of siNGBR RNA sequencing data utilizing transcription factor geneset generated report was performed. Log fold change was compared among significantly enriched transcription factors ($P < 0.05$, FDR q-value<0.25). Comparison between the CCM group and the transcription factors enriched in the NGBR knockdown experimental group does not show a direct correlation. B. Transcription factors CREB1 and ATF2 downregulated in *NGBR* deficient HBMVECs. C. The knockdown of either *CREB1* or *ATF2* does not affect CCM1 and CCM2 expression.

Supplementary Table 1: Chemicals, plasmids, antibodies and siRNAs used in the study

Name	Cat#	Company
Tamoxifen	T5648	Sigma-Aldrich
Evans Blue	E2129	Sigma-Aldrich
FITC-conjugated dextran	FD2000S	Sigma-Aldrich
FITC-conjugated dextran	FD40S	Sigma-Aldrich
Hematoxylin	MHS16	Sigma-Aldrich
alcoholic eosin Y	1024390500	Sigma-Aldrich
VECTASTAIN® Elite ABC-HRP Kit, Peroxidase (Mouse IgG)	PK-6102	Vector Laboratory
Iron Stain Kit	HT20-1KT	Sigma-Aldrich
TritonX-100	10789704001	Sigma-Aldrich
Tween-20	P1379	Sigma-Aldrich
Paraformaldehyde	158127	Sigma-Aldrich
collagenase II	17101015	Thermo Fisher Scientific
DNase I	10104159001	Sigma-Aldrich
Percoll	P1644	Sigma-Aldrich
collagenase/dispase	SCR139	Sigma-Aldrich
SimpleChIP (R) Plus Kits	9004&9005	Cell Signaling
**plasmids**		
psPAX2	#12260	Addgene
pVSV-G	#12259	Addgene
pWPXLD	#12258	Addgene
**antibodies**		
Isolectin B4	I21412	Thermo Fisher Scintific
DAPI	D9542	Sigma-Aldrich
CD31 antibody	AP436PU-N	Acris
CD31 antibody	550274	BD pharmacy
phos-MLC antibody	3671S	Cell Signaling
VE-cadherin antibody	550548	BD pharmacy
VE-cadherin antibody	2500S	Cell Signaling
Claudin-5 antibody	34-1600	Thermo Fisher Scintific
ZO-1 antibody	40-2200	Thermo Fisher Scintific
GFP antibody	50430-2-AP	Proteintech
GFP antibody	GFP-1010	Aves
RhoA antibody	2117S	Cell Signaling
NGBR antibody	ab168351	Abcam
GAPDH antibody	60004-1-Ig	Proetintech
ACTIN antibody	66009-1-Ig	Proetintech
CCM1 antibody	ab196025	Abcam

CCM2 antibody	26270-1-AP	Proteintech
CCM3 antibody	66440-1-Ig	Proteintech
HBO1 antibody	Ab70183	Abcam
HBO1 antibody	58418	Cell Signaling
GCN5 antibody	3305S	Cell Signaling
Histone H3 (D1H2) XP® Rabbit mAb	4499	Cell Signaling
Acetyl-Histone H3 (Lys9) (C5B11) Rabbit mAb	9649	Cell Signaling
Acetyl-Histone H3 (Lys14) (D4B9) Rabbit mAb	7627	Cell Signaling
Acetyl-Histone H3 (Lys18) (D8Z5H) Rabbit mAb	13998	Cell Signaling
Acetyl-Histone H3 (Lys27) (D5E4) XP® Rabbit mAb	8173	Cell Signaling
Histone H4 (L64C1) Mouse mAb	2935	Cell Signaling
Acetyl-Histone H4 (Lys5) (D12B3) Rabbit mAb	8647	Cell Signaling
Acetyl-Histone H4 (Lys8) Antibody	2594	Cell Signaling
Acetyl-Histone H4 (Lys12) (D2W6O) Rabbit mAb	13944	Cell Signaling
SREBP-1c antibody	ab28481	Abcam
**siRNAs**		
CCM1 siRNA	sc-43884	Santa Cruz Biotechnology
CCM2 siRNA	sc-62594	Santa Cruz Biotechnology
HBO1 siRNA	forward sequence: GGCUAAGCCAGAGUUCUCA; reverse sequence: UGAGAACUCUGGCUUAGCC	Generated from IDT
GCN5 siRNA	sc-37946	Santa Cruz Biotechnology
NGBR siRNA	forward sequence: GGAAAUACAUAGACCUACA; reverse sequence: UGUAGGUCUAUGUAUUUCC	Generated from IDT

SREBP-1c siRNA	sc-36557	Santa Cruz Biotechnology

Supplementary Table 2: Oligonucleotide sequences of primers used

**RT-PCR primers**	Forward (5′-3′)	Reverse (5′-3′)
Ngbr (mouse)	TCCTACATTAGCGTCTACGACC	GCTCTCACAATATCCGCTTTTCC
NGBR (human)	TGCCAGTGAGATGCCCAGAAGCAA	TGATGTGCCAGGGAAGAAAGCCTA
Ccm1 (mouse)	GAAAGACGCCATTAACAAGCC	CCGCATTCCCTCCATTATCTG
Ccm2 (mouse)	AGAAAGCCCATGAGAAGGTG	CGGGAATGGATGTGAACTGACC
CCM1 (human)	CTGTAAGAACATGCGCTGAAG	TCCATCGTACCTGTTACCAAAC
CCM2 (human)	AGAAAGCCCATGAGAAGGTG	CCTGGTATGGACGTTAACTGAC
Hbo1 (mouse)	CCCGCTGTATCATAACCTCTC	AGCCACCTTTTCCTTATACCG
HBO1 (human)	AGCCCTTCCTGTTCTATGTTATG	CATAGCCCTGTCTCATGTACTG
GAPDH (human)	TGGACAGTCAGCCGCATCTTCTTT	ACCAAATCCGTTGACTCCGACCTT
Gapdh (mouse)	CTGGAGAAACCTGCCAAGTA	TGTTGCTGTAGCCGTATTCA
ACTIN (human)	TTCTACAATGAGCTGCGTGTGGCT	TAGCACAGCCTGGATAGCAACGTA
Actin (mouse)	GGCTGTATTCCCCTCCATCG	CCAGTTGGTAACAATGCCATGT
HDAC1 (human)	GAGATGACCAAGTACCACAGC	TGACAGAACTCAAACAGGCC
HDAC2 (human)	TGACAAACCAGAACACTCCAG	CTTCTCCATCTTCATCTCCACTG
HDAC3 (human)	GGACTTCTACCAACCCACG	CAGCACGAGTAGAGGGATATTG
HDAC4 (human)	ACAAGGAGAAGGGCAAAGAG	GCGTTTTCCCGTACCAGTAG
HDAC5 (human)	TCACCGCAAAACTCCTACAG	AGTTCCCGTTGTCATAGCG
HDAC6 (human)	TTCAACTCTGTGGCTGTGG	GCAGGGACACATATAGCACAC
HDAC7 (human)	GCAGATCATTCAACAGCCATG	TTGGTAGAAGGTTTGCTGGG
HDAC8 (human)	AATTAACTGGTCTGGAGGTG	TGCAGATCCAAATCCACGTAG
HDAC9 (human)	ACACATTACCAGGAGCACAAG	CAACATTTCCATCCTTCCGC
HDAC10 (human)	AGAAACACGGGCTACACAG	GGTGCCAGGAGAAGTAAAGG
HDAC11 (human)	GTTTCTGTTTGAGCGTGTGG	GGTAGATGTGGCGGTTGTAG
SIRT1 (human)	CCCTCAAAGTAAGACCAGTAGC	CACAGTCTCCAAGAAGCTCTAC
*SIRT*2 (human)	ACCTTCTACACATCACACTGC	GACGATATCAGGCTTCACCAG
SIRT3 (human)	TCATGGAACCTTTGCCTCTG	GCTCCCCAAAGAACACAATG
SIRT4 (human)	TCGGAAAGCTGTACTGGTTG	TCTGTTCCCCACAATCCAAG
SIRT5 (human)	GCCTCCCGCAGAATTGGTA	AGAGGTCGCATCAGGGTTTG
SIRT6 (human)	AGGATGTCGGTGAATTACGC	GAAGACTGCCAGACCAGC
SIRT7 (human)	AGAAAGGGAGAAGCGTTAGTG	GAGCCCGTCACAGTTCTG
KAT1 (human)	ACTCCATTTCAAGGTCAAGGC	CTTCACAAGCACAAAGTCTCG
GCN5 (human)	TCTCTACTTCCTCACCTACGC	ATTCAGCTCACACTCCATCAG
PCAF (human)	GAAGAGAACAGAAGCTCCAGG	GCAATTGGTAAAGACTCGCTG
CREBBP (human)	CAACCCCAAAAGAGCCAAAC	GGTTCCCACTGTTTAAAAGGC
EP300 (human)	GACCAGACTACAGAAGCAGAAC	ACTGCCACGGATCATACTTG
KAT5 (human)	CATCGTGGGCTACTTCTCC	CCTGTTTTCCCTTCCACTTTG
KAT6A (human)	ACATCACTTCCACACTCCAC	CATCTACAGGTCGCAAATTCAG
KAT6B (human)	GACAAACAGAGGAAGAGGAAGG	TCGGGATTGTCTTTACTGCC
KAT8 (human)	CAAGATCACTCGCAACCAAAAG	TGTCCACATACTTCACCTTGG
SREBP-1c (human)	CAACACAGCAACCAGAAACTC	CTCCACCTCAGTCTTCACG
**ChIP assay primers**	Forward (5′-3′)	Reverse (5′-3′)
CCM1 promotor	ATACAGGGGAGCGCTCCATTC	TAAAAGTGCTCTGCAGGGCTG
CCM2 promotor	CTTGCAGTGAGCCGAGATC	GCACAGCTAGAATGTAAACTGTG

HBO1 promotor	GTACTGCATTCCCCACTTC	CCATTCTCCAGAAGCTGCAG

Full unedited gel for Figure 3D

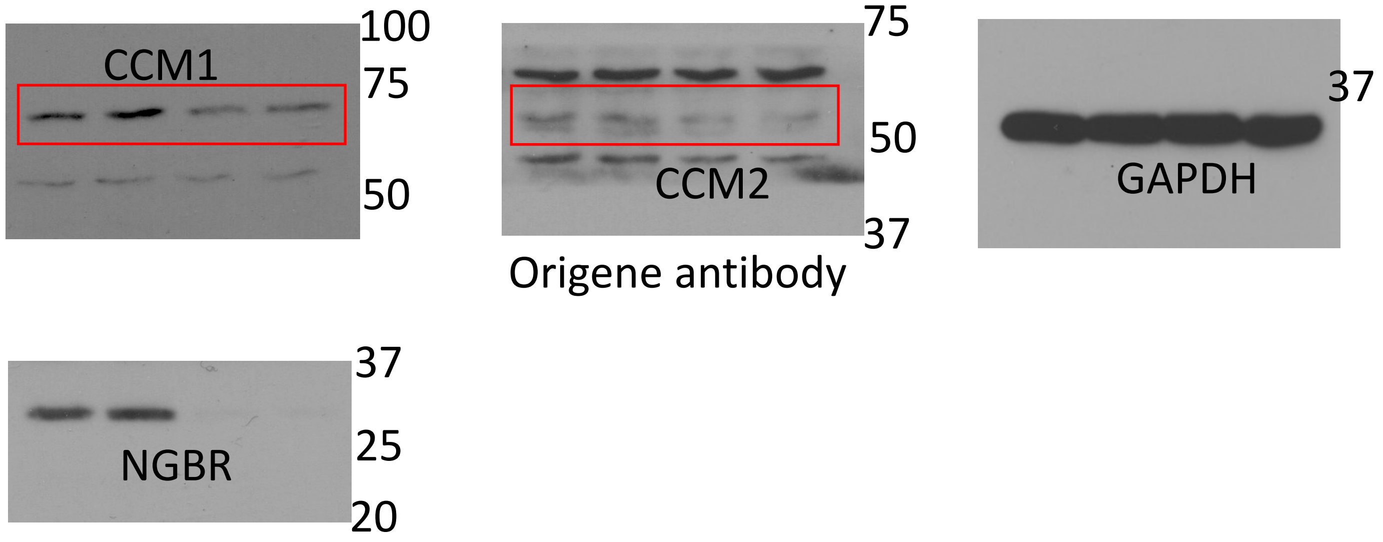

Full unedited gel for Figure 3H

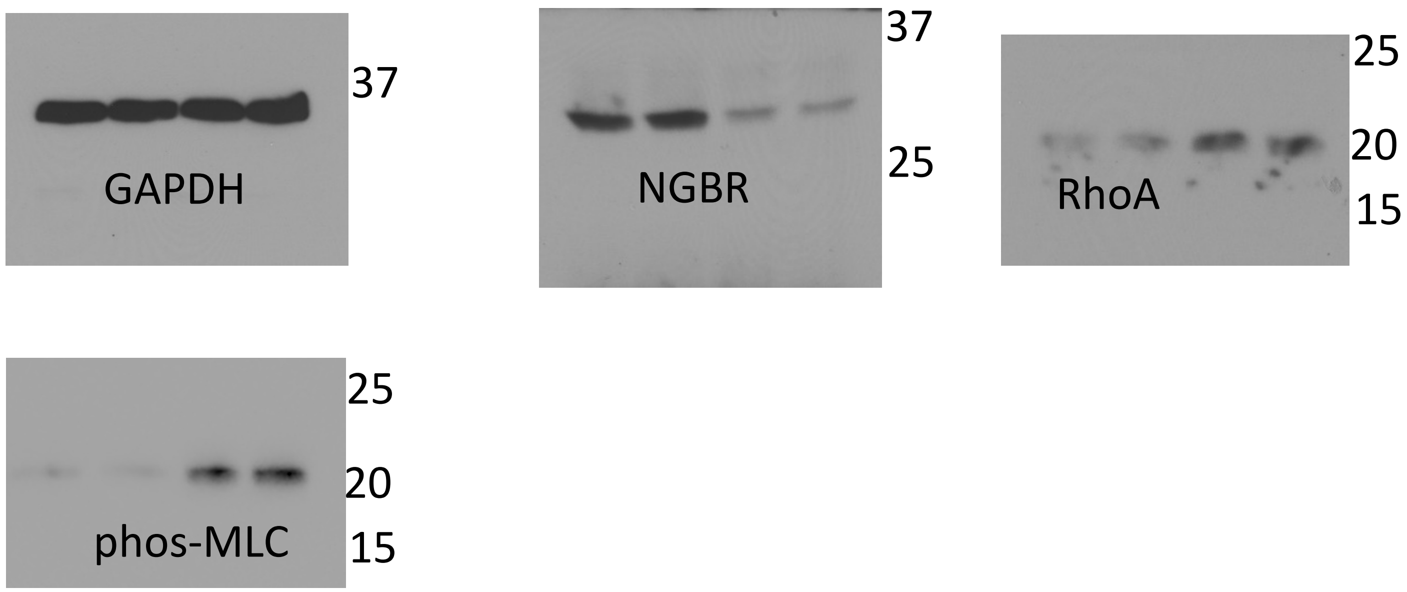

Full unedited gel for Figure 4D

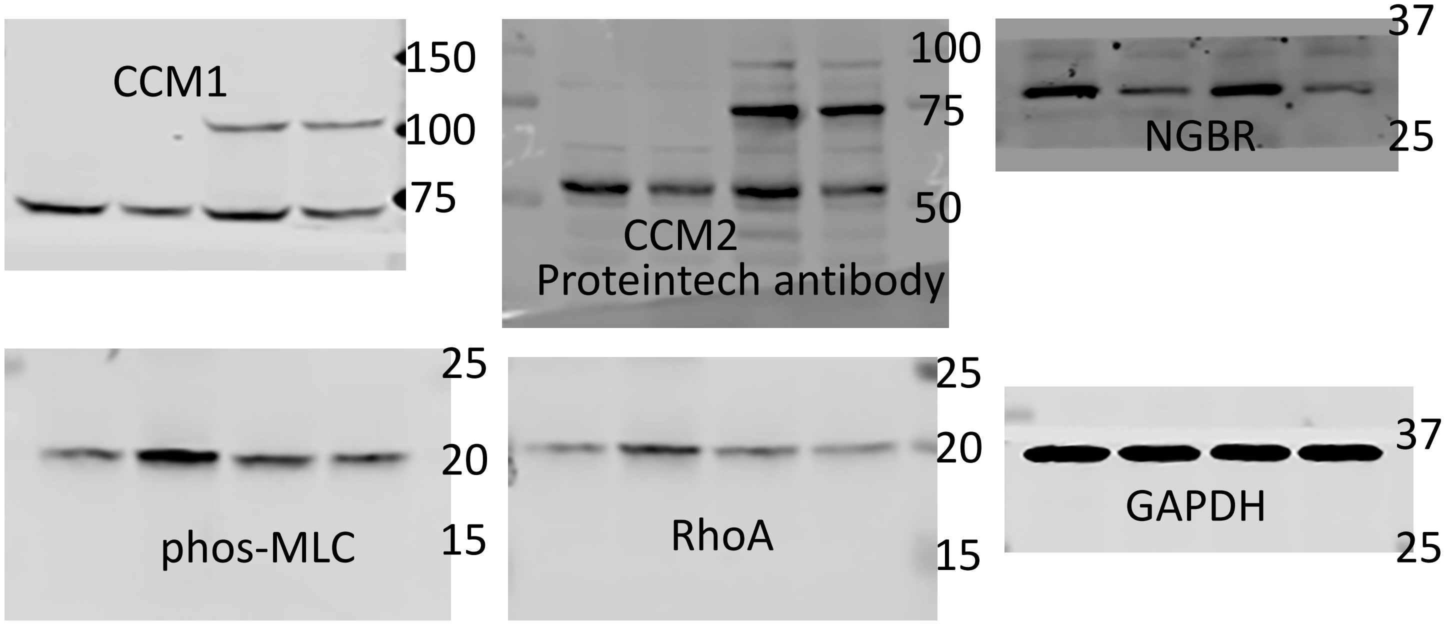

Full unedited gel for Figure 5B

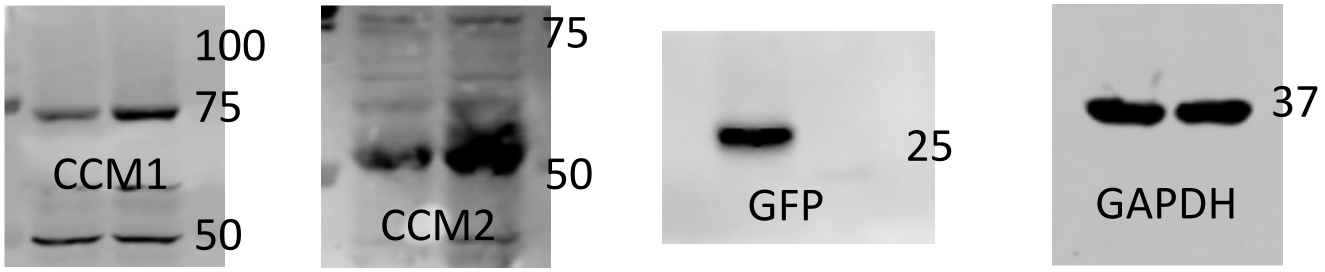

Full unedited gel for Figure 5G

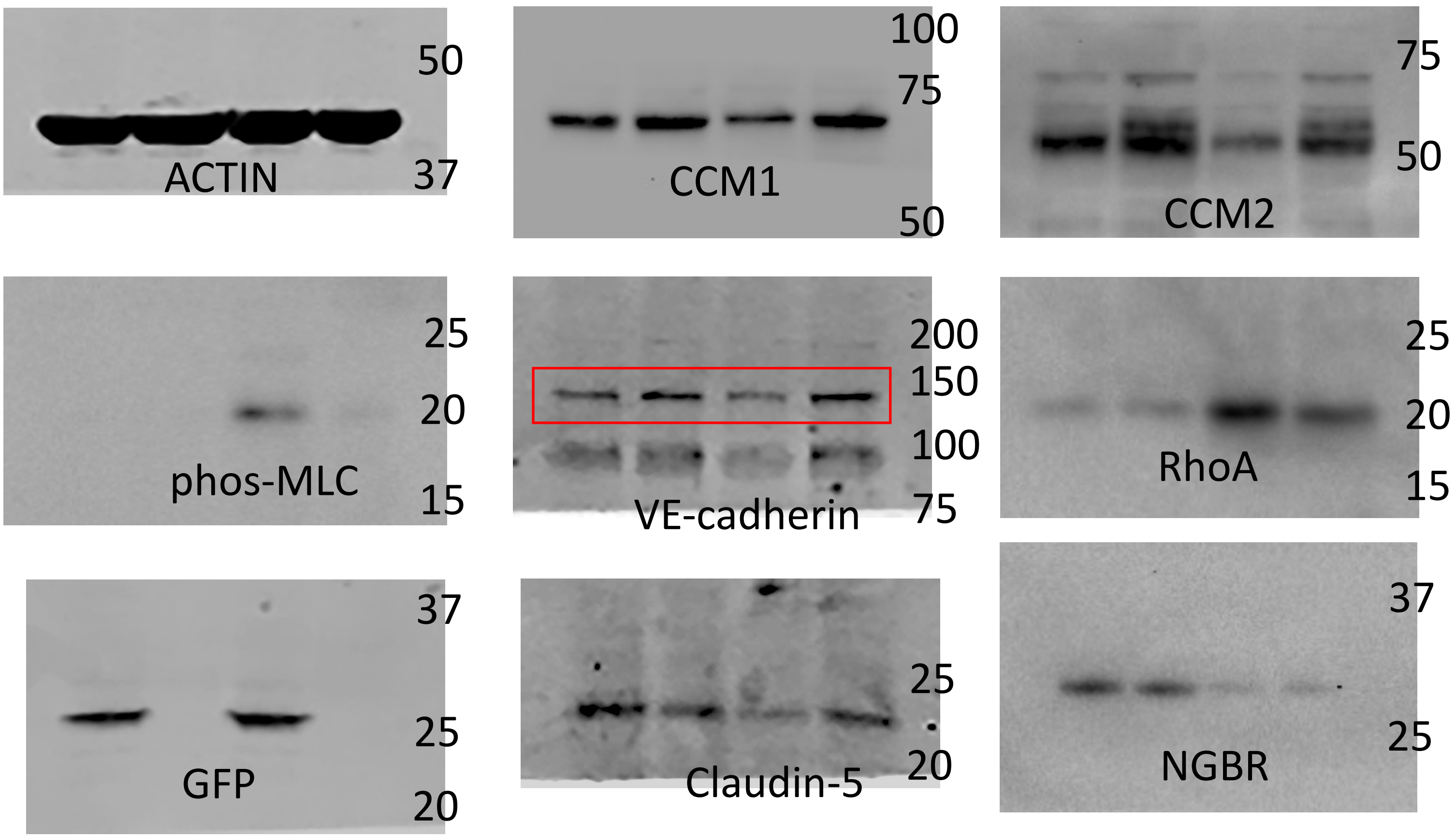

Full unedited gel for Figure 6D

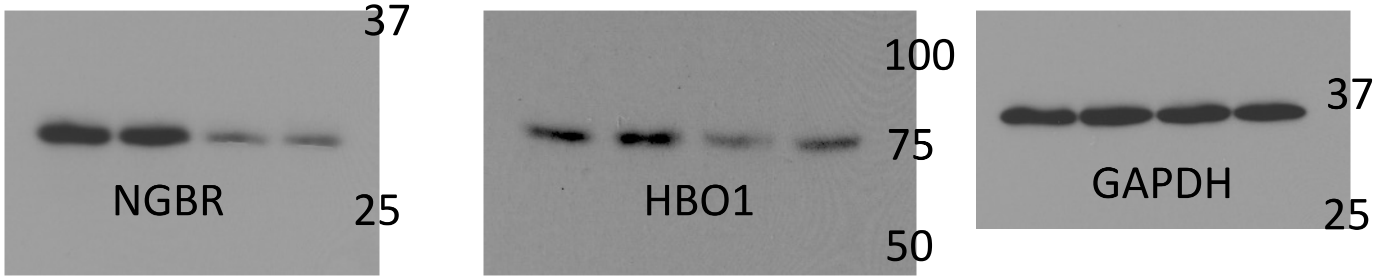

Full unedited gel for Figure 6H

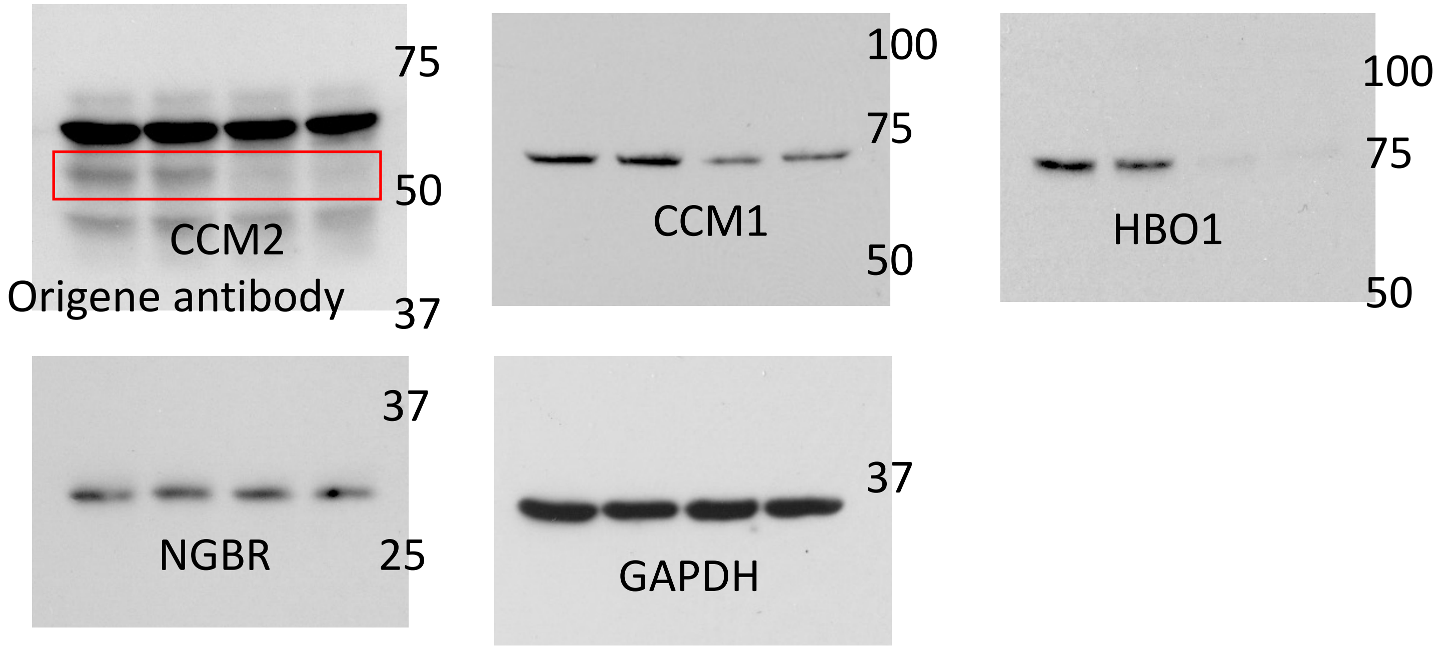

Full unedited gel for Figure 6J

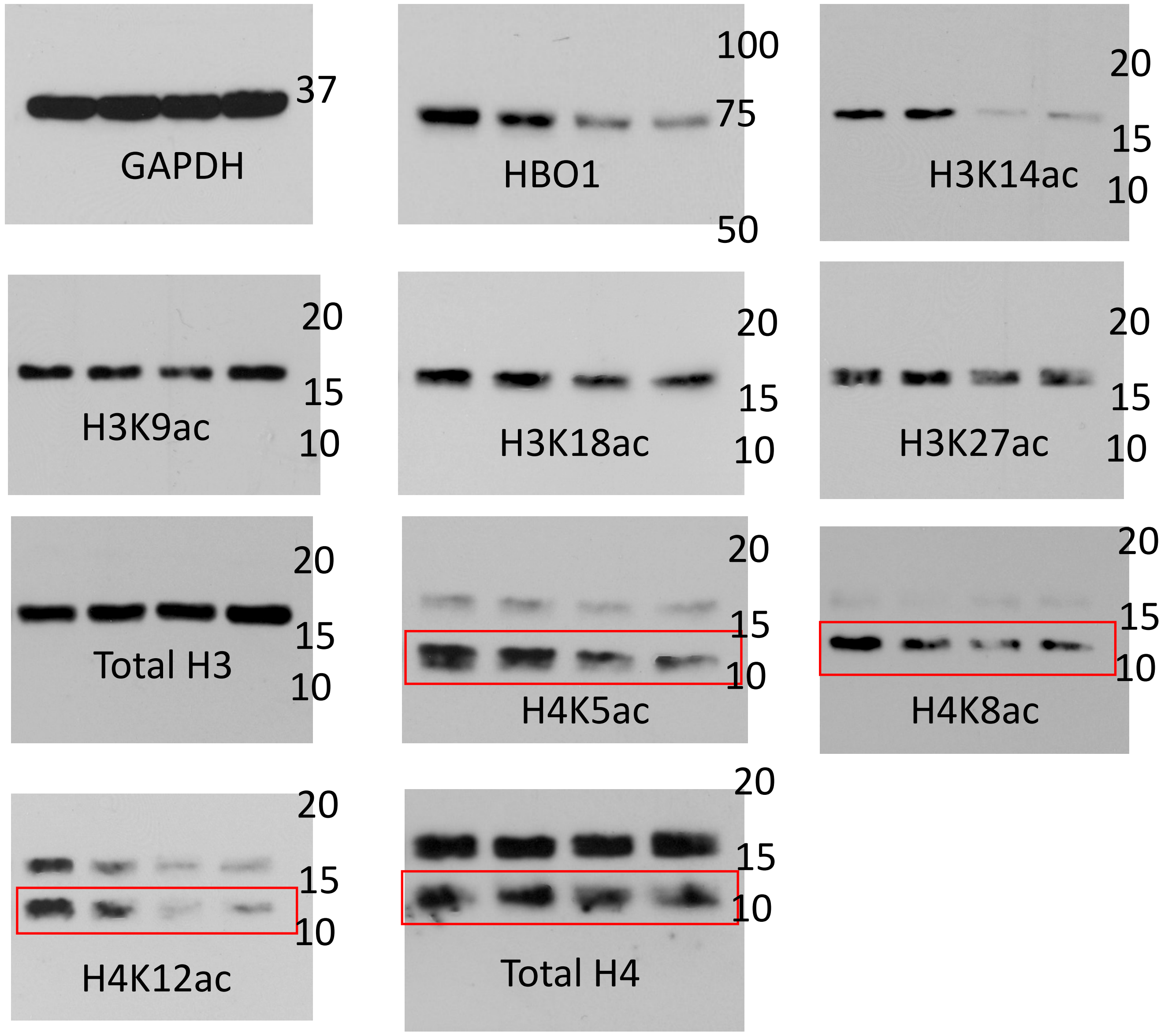

Full unedited gel for Figure 6M

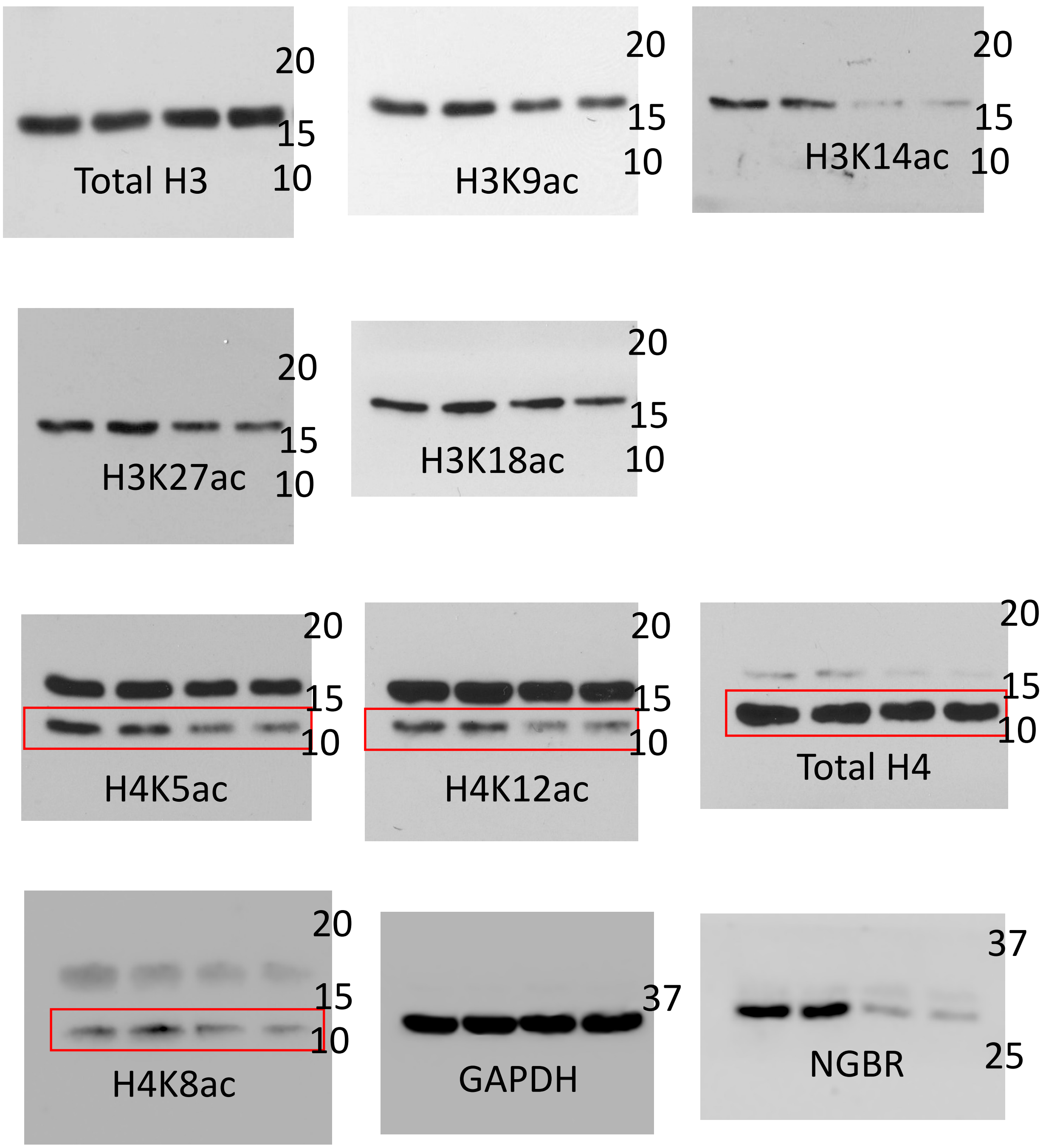

Full unedited gel for Figure 7H

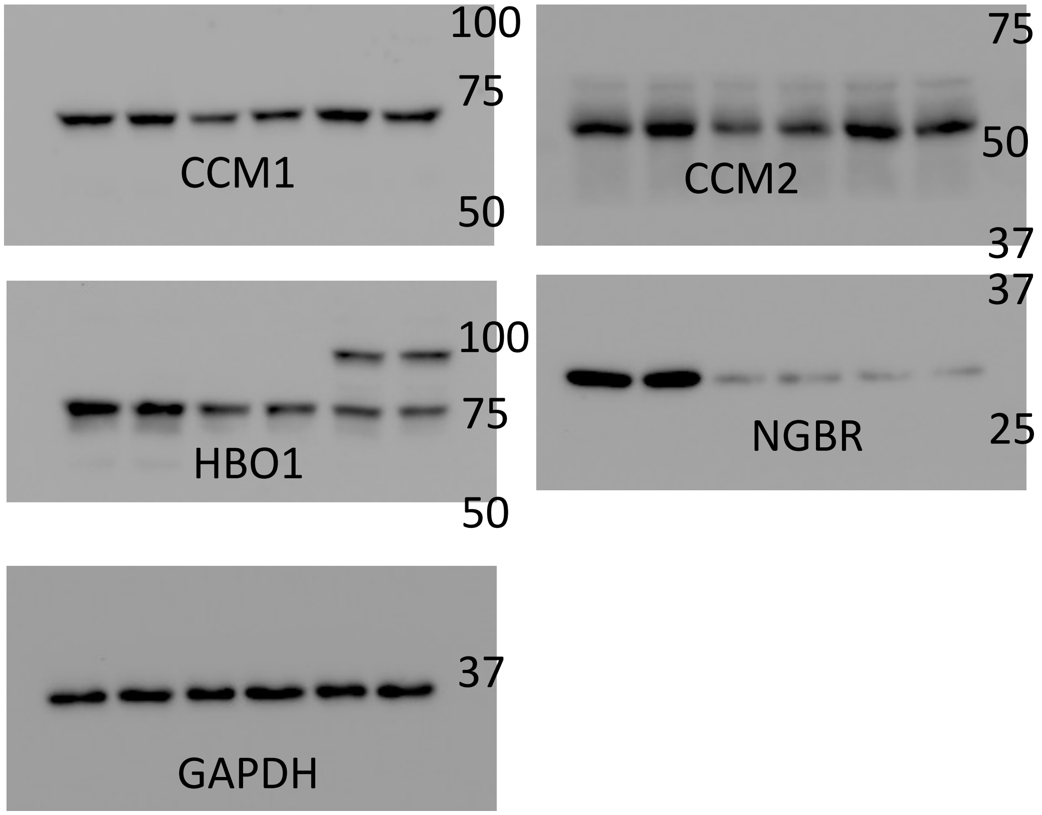

Full unedited gel for Supplementary Figure 2C

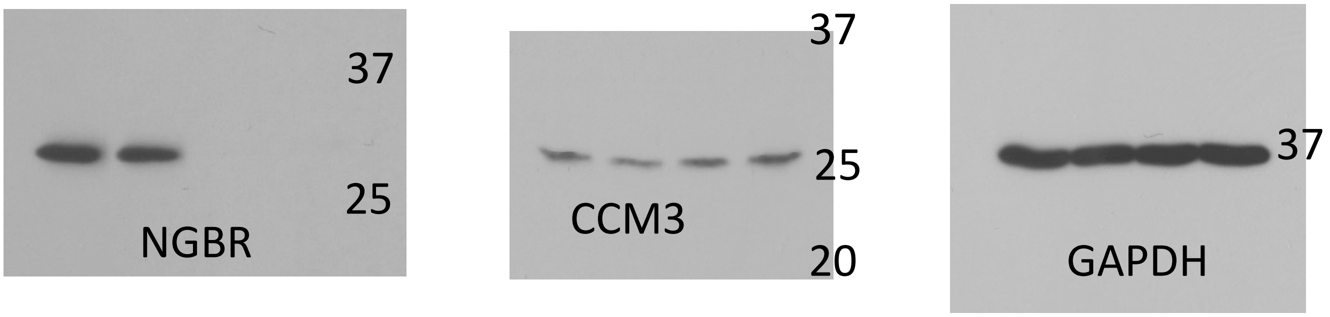

Full unedited gel for Supplementary Figure 3C

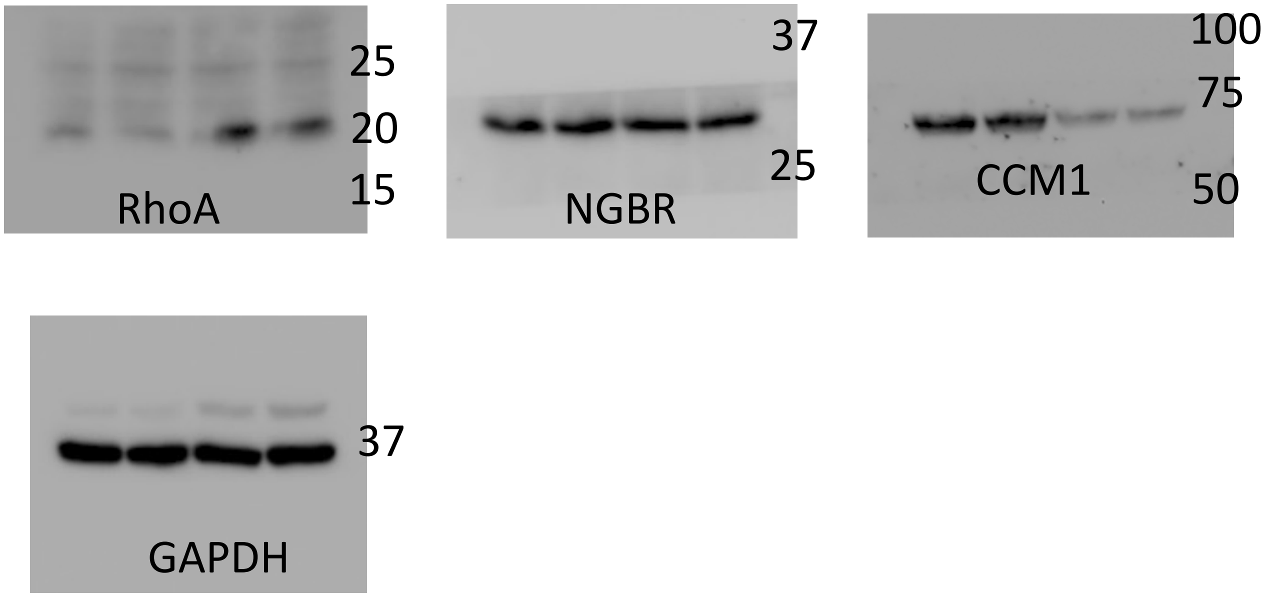

Full unedited gel for Supplementary Figure 3D

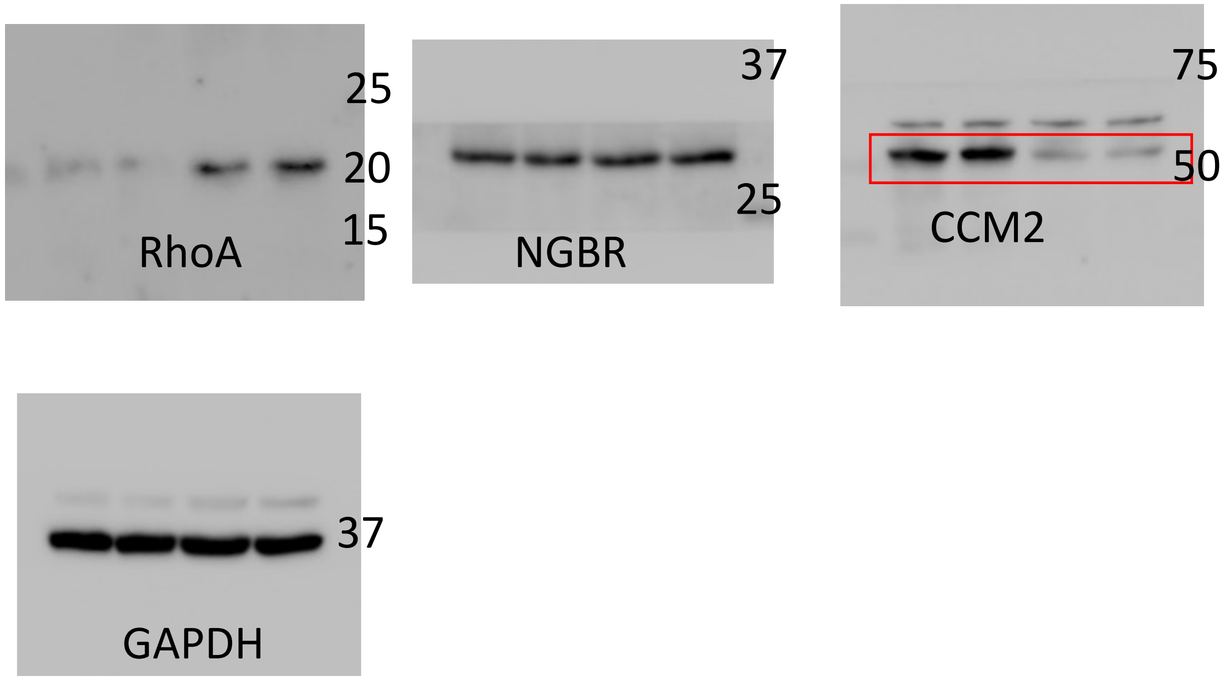

Full unedited gel for Supplementary Figure 4A

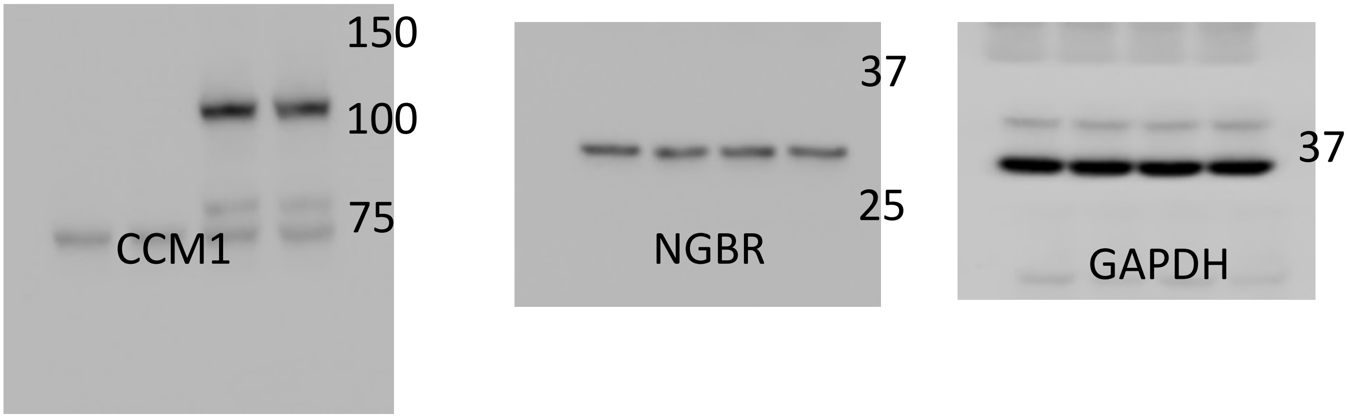

Full unedited gel for Supplementary Figure 4B

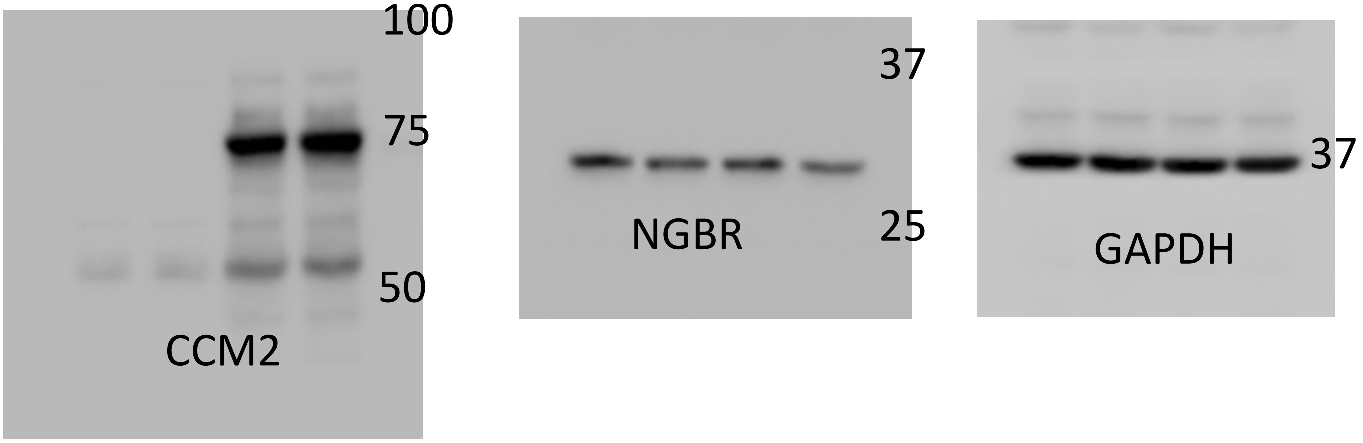

Full unedited gel for Supplementary Figure 7C

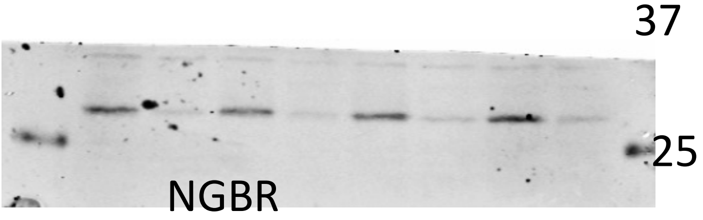

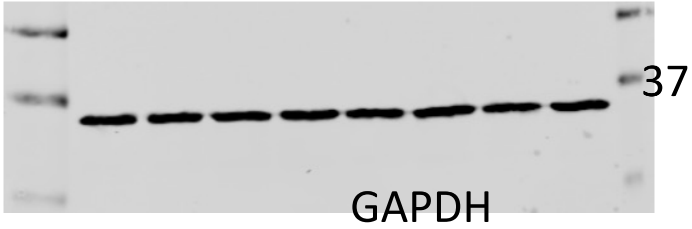

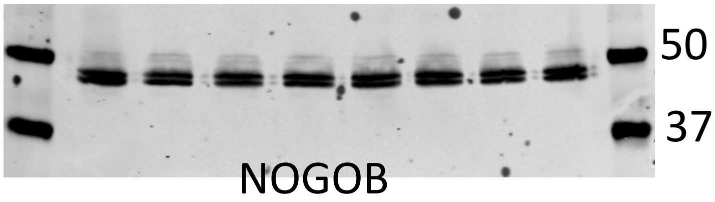

Full unedited gel for Supplementary Figure 8D

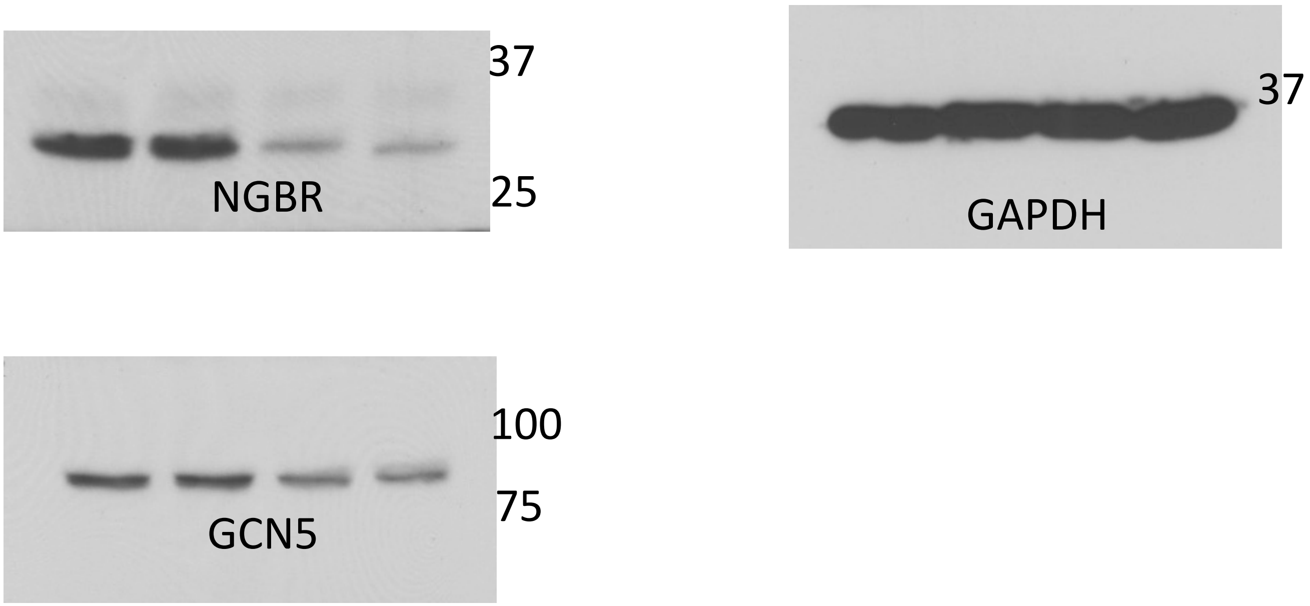

Full unedited gel for Supplementary Figure 8E

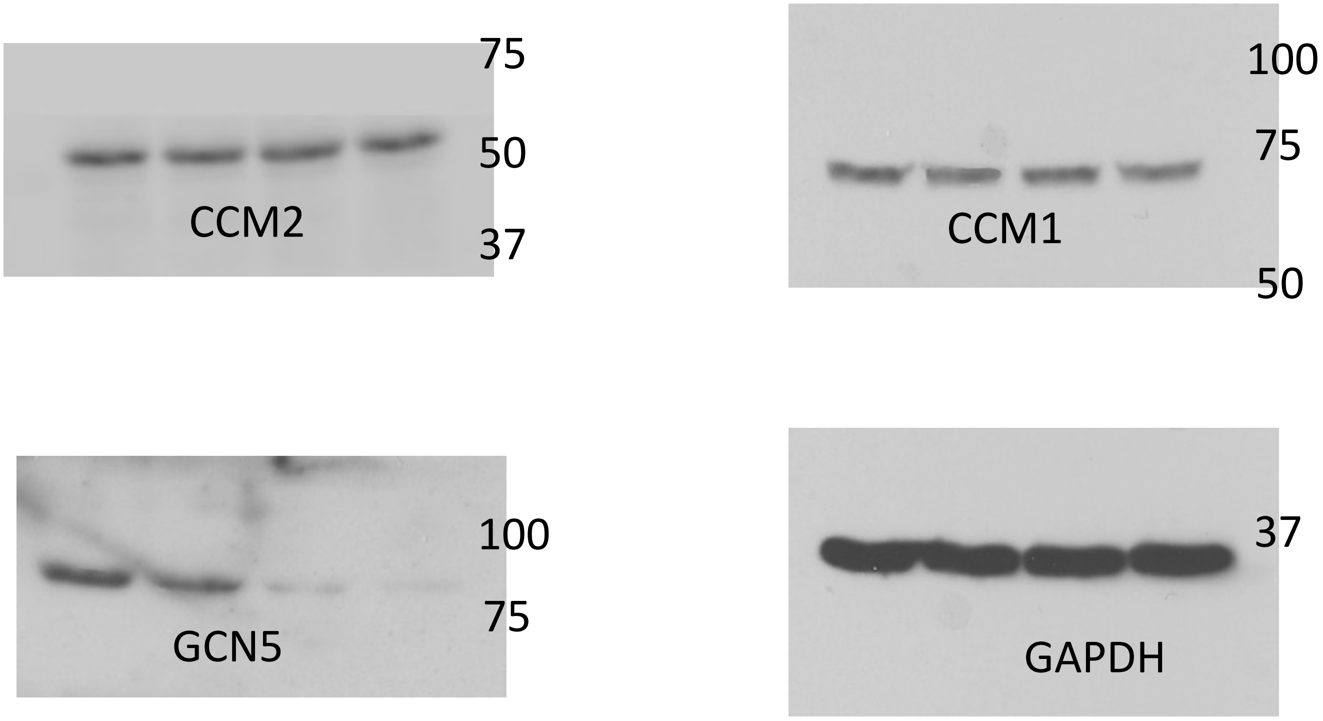

Full unedited gel for Supplementary Figure 9A

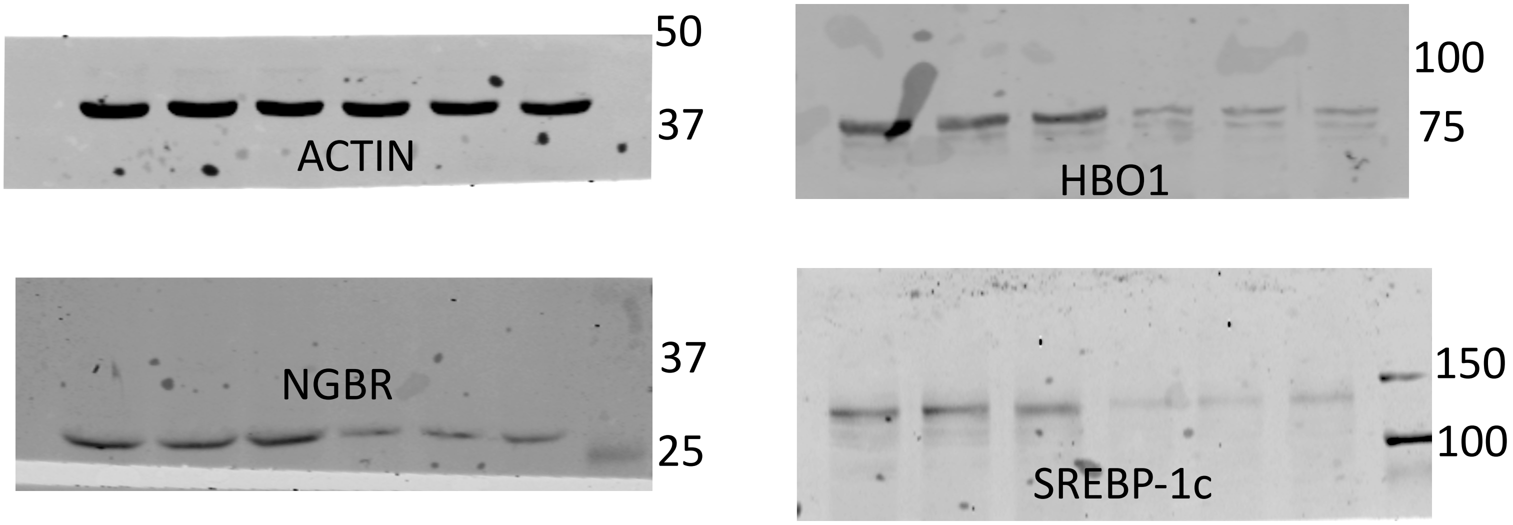

Full unedited gel for Supplementary Figure 9C

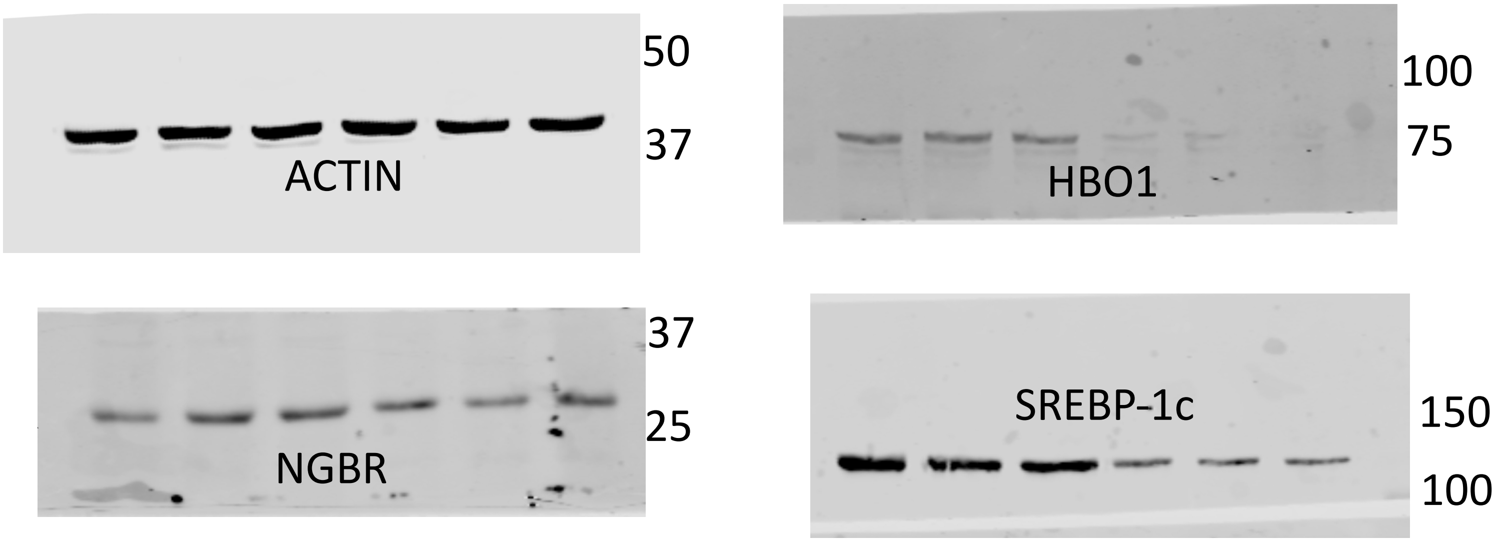

Full unedited gel for Supplementary Figure 11B

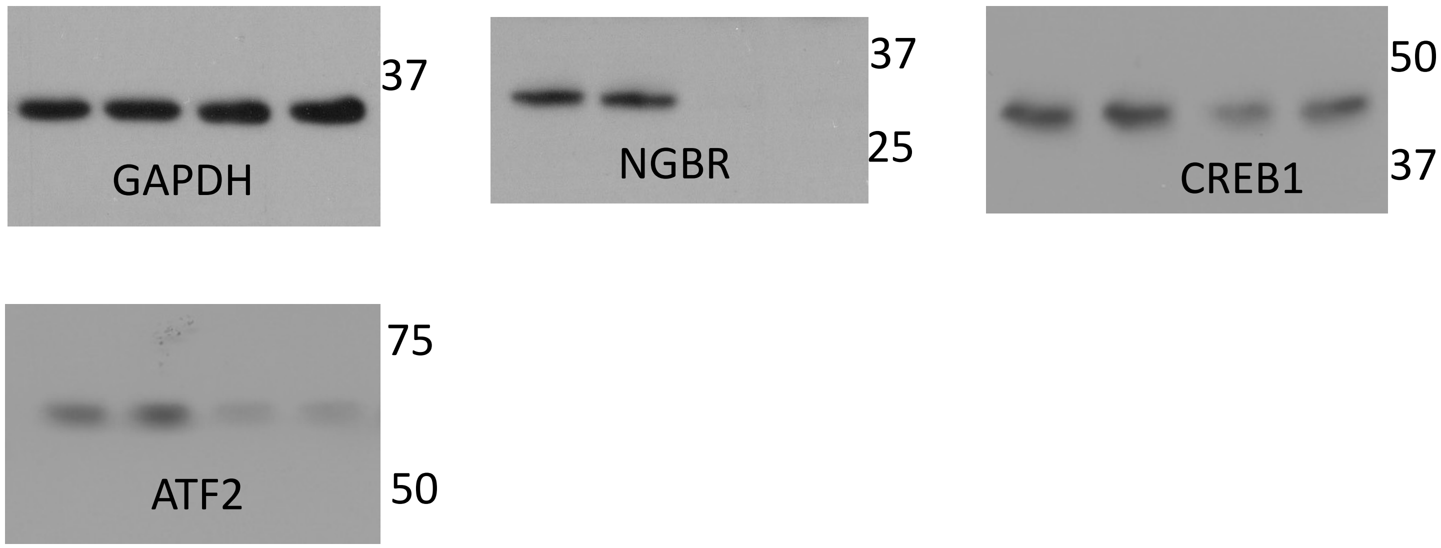

Full unedited gel for Supplementary Figure 11C

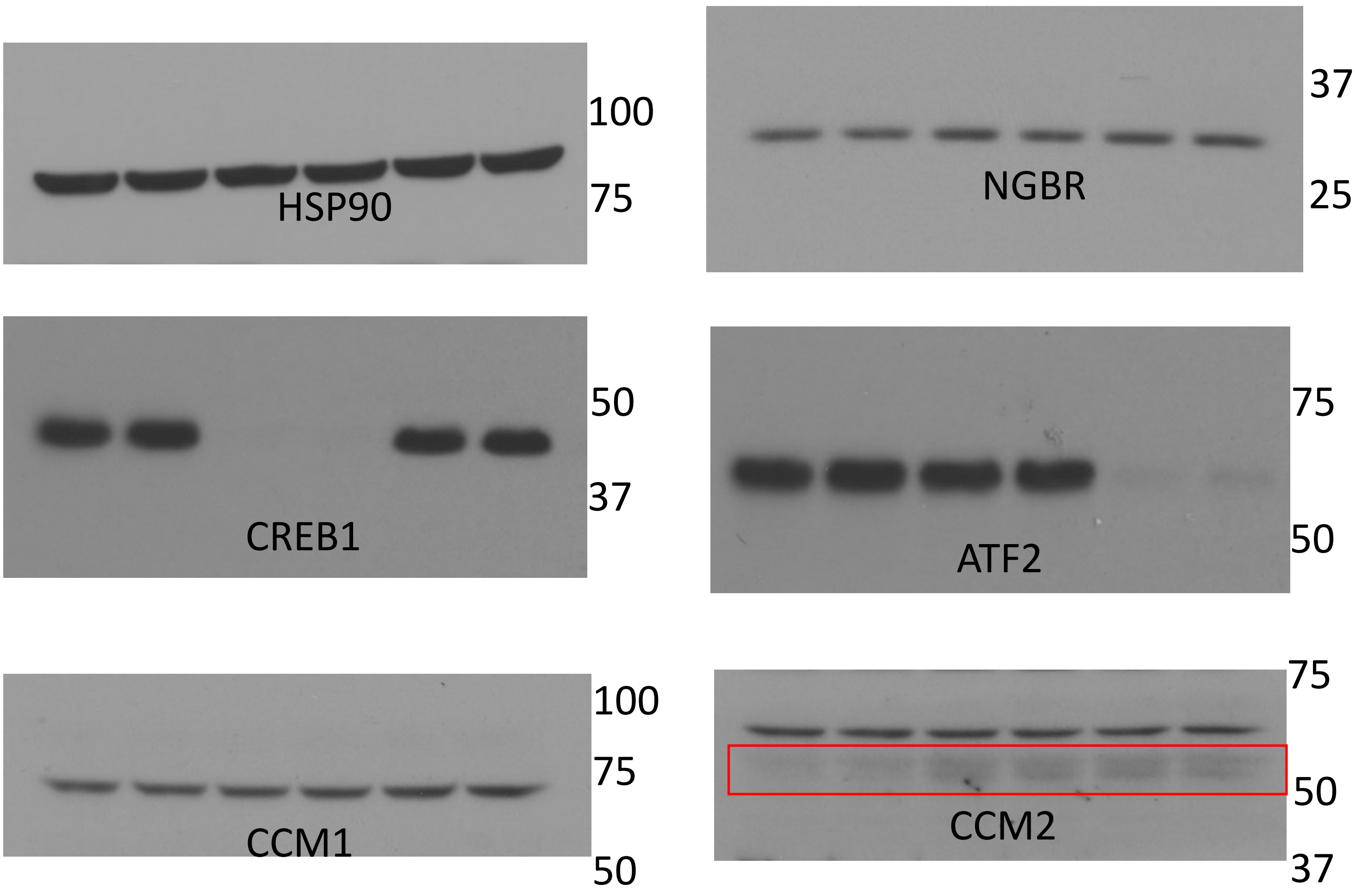

Supplement: Supplemental data [file jci-132-151382-s032.pdf]
